# Uncertainty Quantification of Regional Cardiac Tissue Properties in Arrhythmogenic Cardiomyopathy Using Adaptive Multiple Importance Sampling

**DOI:** 10.3389/fphys.2021.738926

**Published:** 2021-09-30

**Authors:** Nick van Osta, Feddo P. Kirkels, Tim van Loon, Tijmen Koopsen, Aurore Lyon, Roel Meiburg, Wouter Huberts, Maarten J. Cramer, Tammo Delhaas, Kristina H. Haugaa, Arco J. Teske, Joost Lumens

**Affiliations:** ^1^Department of Biomedical Engineering, Cardiovascular Research Institute Maastricht, Maastricht University, Maastricht, Netherlands; ^2^Division Heart and Lungs, Department of Cardiology, University Medical Center Utrecht, Utrecht, Netherlands; ^3^Department of Cardiology, Oslo University Hospital, University of Oslo, Oslo, Norway

**Keywords:** arrhythmogenic right ventricular cardiomyopathy, speckle-tracking echocardiography, deformation imaging, cardiac computational model, adaptive multiple importance sampling

## Abstract

**Introduction:** Computational models of the cardiovascular system are widely used to simulate cardiac (dys)function. Personalization of such models for patient-specific simulation of cardiac function remains challenging. Measurement uncertainty affects accuracy of parameter estimations. In this study, we present a methodology for patient-specific estimation and uncertainty quantification of parameters in the closed-loop CircAdapt model of the human heart and circulation using echocardiographic deformation imaging. Based on patient-specific estimated parameters we aim to reveal the mechanical substrate underlying deformation abnormalities in patients with arrhythmogenic cardiomyopathy (AC).

**Methods:** We used adaptive multiple importance sampling to estimate the posterior distribution of regional myocardial tissue properties. This methodology is implemented in the CircAdapt cardiovascular modeling platform and applied to estimate active and passive tissue properties underlying regional deformation patterns, left ventricular volumes, and right ventricular diameter. First, we tested the accuracy of this method and its inter- and intraobserver variability using nine datasets obtained in AC patients. Second, we tested the trueness of the estimation using nine *in silico* generated virtual patient datasets representative for various stages of AC. Finally, we applied this method to two longitudinal series of echocardiograms of two pathogenic mutation carriers without established myocardial disease at baseline.

**Results:** Tissue characteristics of virtual patients were accurately estimated with a highest density interval containing the true parameter value of 9% (95% CI [0–79]). Variances of estimated posterior distributions in patient data and virtual data were comparable, supporting the reliability of the patient estimations. Estimations were highly reproducible with an overlap in posterior distributions of 89.9% (95% CI [60.1–95.9]). Clinically measured deformation, ejection fraction, and end-diastolic volume were accurately simulated. In presence of worsening of deformation over time, estimated tissue properties also revealed functional deterioration.

**Conclusion:** This method facilitates patient-specific simulation-based estimation of regional ventricular tissue properties from non-invasive imaging data, taking into account both measurement and model uncertainties. Two proof-of-principle case studies suggested that this cardiac digital twin technology enables quantitative monitoring of AC disease progression in early stages of disease.

## Introduction

Computational models of the cardiovascular system are widely used to simulate cardiac (dys)function and related clinical application of therapies for cardiac disease (Niederer et al., 2019). Various attempts to generate a digital twin of the human heart have been made (Corral-Acero et al., 2020). Previously, we proposed a framework to create a digital twin (van Osta et al., 2020) for quantification of the disease substrate underlying abnormal tissue deformation in patients with arrhythmogenic cardiomyopathy (AC) (van Osta et al., 2021).

Inheritable AC primarily affects the right ventricle (RV) and predisposes to ventricular arrhythmias and sudden cardiac death in young individuals (Thiene et al., 1988; Basso et al., 2009). Therefore, early disease detection is important. We previously determined an *in silico* disease substrate with decreased regional RV contractility and compliance, with the potential to predict disease progression on a patient-specific level (van Osta et al., 2021). This method was, however, not able to include uncertainty present in both measurement and model.

Uncertainty will inevitably play a role in comparing estimated properties and thus Bayesian inference methods should be used to estimate the posterior distribution of model parameters, rather than only providing point estimates. Cardiovascular computational models are in general complex, meaning that the posterior distribution cannot be calculated analytically. Various techniques have been proposed to solve this problem, in which Markov chain Monte Carlo (MCMC) methods are often used (Schiavazzi et al., 2017; Dhamala et al., 2018; Meiburg et al., 2021). Adaptive multiple importance sampling (AMIS) is an important alternative to MCMC since it enables estimation of the posterior distribution in a model with a relatively high number of input parameters (Cornuet et al., 2012; Bugallo et al., 2017).

In this study, we apply AMIS to quantify parameter uncertainties in digital twins based on echocardiographic deformation imaging. We validate the methodology based on both *in silico* generated virtual data and datasets obtained from patients with AC and mutation positive family-members at risk of developing the disease. Furthermore, we use longitudinal series of echocardiograms in two AC patients to validate clinical applicability of this methodology.

## Materials and Methods

**FIGURE 1:**
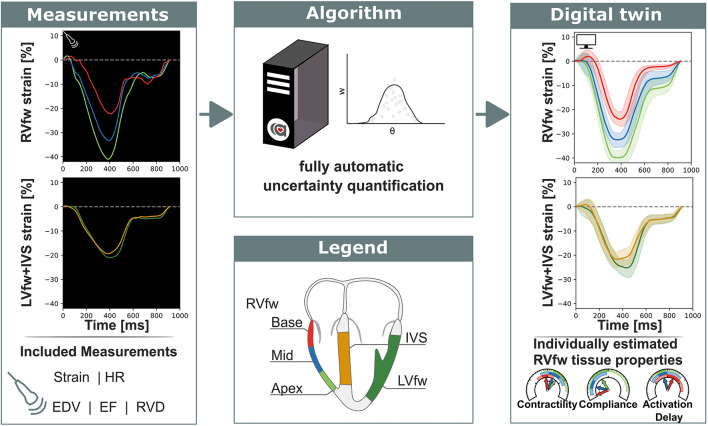
Non-invasive measurements were used as input for a fully automatic automated uncertainty quantification algorithm. This algorithm produced a digital twin based on estimated parameters with accompanying uncertainty. This digital twin can be used to get more insight in the estimated tissue properties. RVfw, right ventricle free wall; LVfw, left ventricle free wall; IVS, inter ventricular septum; HR, heart rate; EDV, end-diastolic volume; EF, ejection fraction; RVD, right ventricular diameter.

This section and Figure 1 elucidate the methodology used to estimate parameters and related uncertainties using the CircAdapt model. First, we elaborate the mathematical basis and implementation of AMIS, which is generally applicable. Secondly, we describe the mathematical problem and introduce the included clinical measurements and the computational model used for the likelihood function. Finally, we explain the simulation protocol. More detailed information is shown in Supplementary Material, including pseudocodes of the algorithm. The source code as well as the virtual patient datasets are available.

### Mathematical Basis of Adaptive Multiple Importance Sampling

We consider an *n*_θ_ -dimensional vector as a set of parameters θ of a numerical model *z* = ℳ(θ). This model ℳ:ℛ^*n*_θ_^→ℛ^*n*_*z*_^ maps the parameter vector to an *n_z*-dimensional vector of modeled data *z*. Measurement uncertainties are included in the likelihood function *p*(*z*|θ) representing the similarity between patient observation and model output. The posterior distribution *p*(θ|*z*) is the probability of having parameters θ given the observation *z* and is given by Bayes’ rule as


(1) $$p\left(\theta |z\right)=\frac{p\left(z|\theta \right)p\left(\theta \right)}{p\,\left(z\right)}\propto p\left(z|\theta \right)p\left(\theta \right),$$

with *p*(θ) the prior knowledge of the parameters and *p*(*z*) the normalizing constant. No prior knowledge of the parameters *p*(θ) is known, so *p*(θ) was assumed to be uniform.

Importance sampling is an algorithm which estimates the posterior distribution *p*(θ|*z*) (Bugallo et al., 2017). The set of samples Θ = {θ∼*q*(θ)} drawn from the proposal distribution *q*(θ) form an empirical estimation of the posterior distribution *p*(θ|*z*) in which each sample is weighted with the sample weight *w* described by


(2) $$w\,\left(\theta \right)\propto \frac{p\left(\theta |z\right)}{q\,\left(\theta \right)}.$$

The weights are normalized such that∑_θ ∈ Θ_*w*(θ) = 1. Importance sampling is most effective when the proposal distribution *q*(θ) is close to the posterior distribution *p*(θ|*z*) such that variance in weight of the samples is small and the effective sample size is close to the actual sample size. Since no information was available on the posterior distribution, we used adaptive importance sampling in which the proposal distribution is iteratively updated to better describe the posterior distribution (Bugallo et al., 2017).

**FIGURE 2:**
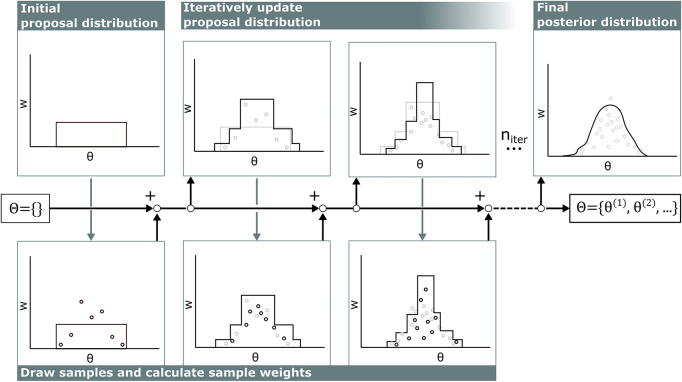
Visualization of adaptive multiple importance sampling. In the first iteration, samples **θ** are drawn from a uniform distribution and stored in the sample set **Θ**. For each sample, the corresponding sample weight **w** is calculated. Then, based on all previous samples **θ** in the sample set **Θ** and corresponding sample weight **w**, the next proposal distribution is defined and new samples are added to the sample set **Θ**. This iterates ****n**_**i***ter*_** times.

The computational cost of calculating the likelihood *p*(*z*|θ) in cardiovascular models is relatively high compared to the cost of calculating the probability density function of the proposal distribution *q*(θ), so the samples from all previous iterations were included in defining the proposal distribution *q*(θ) to optimally recycle past simulations following the AMIS (see Figure 2) (Cornuet et al., 2012).

Each iteration in this algorithm consists of two stages. First, samples are drawn from the proposal distribution and weights of all samples are updated. Second, the proposal distribution is updated based on the new sample weights.

#### Draw Samples and Calculate Sample Weights

At the start of each iteration *i*, 100 samples are drawn from the current proposal distribution π_*i*_(θ). Samples are drawn without statistical dependencies between parameters, which may result in non-physiological combinations of parameters. For example, the model is not parameterized for a low contractile heart to be able to supply a high cardiac output (CO) and is therefore likely to become numerically instable. To circumvent this, only a small uniform distribution around the reference is used as initial proposal distribution *q*_0_(θ). AMIS will increase and decrease the search area of the proposal distribution and will move this to the area of interest in which physiological samples will be drawn close to the desired posterior distribution.

Each iteration, the weights are updated based on the proposal function and likelihood (Equation 2). The probability density function of all previous proposal distributions is given by the sum of all individual proposal distributions


(3) $$q_{i}\,\left(\theta \right)=\frac{1}{N_{s\,a\,m\,p\,l\,e\,s}}\,\sum_{i=0}^{n_{i\,t\,e\,r}-1}n_{s\,a\,m\,p\,l\,e\,s,i}\cdot \pi_{i}\,\left(\theta \right),$$

with *n*_*samples, i*_ the number of samples in iteration *i* and $N_{s\,a\,m\,p\,l\,e\,s}=\sum_{i=0}^{n_{i\,t\,e\,r}-1}n_{s\,a\,m\,p\,l\,e\,s,i}$ the total number of samples. Samples drawn from poorly performing proposal distributions are eliminated through the erosion of their low weights (Cornuet et al., 2012).

The likelihood function is defined based on the normalized dimensionless summed squared error X()^2^. This X(θ)^2^ is problem dependent and the X^2^ used in this study is described in section “Likelihood Function.” We assumed a non-informative uniform prior and neglected all interactions between individual errors. Furthermore, annealed adaptive importance sampling (Li and Lin, 2015) was used to prevent the algorithm from premature convergence (Černý, 1985;, Neal 2001), resulting in a likelihood


(4) $$p\left(z|\theta ,T_{i}\right)\propto e^{-\frac{\text{X}\,{\left(\theta \right)}^{2}}{T_{i}}},$$

in which *T*_*i*_ = 1 in each iteration *i* and represents the annealing temperature. This method is included to control convergence rate, thereby improving global search capabilities and limiting premature convergence toward local minima. The initial temperature is set to *T*_*max*_ = 10, and decreases each iteration *i* such that


(5) $$T_{i+1}=\left\{ \begin{array}{c} \begin{array}{cc} \min\left(10,T_{i}+{\text{X}}_{o\,p\,t}^{2}\right) & \text{if}\,{\text{X}}^{2}\,\mathrm{is}\,\mathrm{improved} \end{array}\\ \begin{array}{cc} \max\left(1,0.8\cdot T_{i}\right) & \text{else} \end{array} \end{array}\right.$$

with ${\text{X}}_{o\,p\,t}^{2}$ the difference between the old and new X^2^ of the best sample.

#### Update Proposal Distribution

Each iteration, the proposal distribution is updated based on all drawn samples in the sample set Θ and its corresponding weight *w*. Details on the definition of the proposal distribution are shown in Supplementary Material 1.1. In the updated proposal distributions, samples were drawn along the principal component axes of the weighted sample set Θ.

This protocol ran for at least 500 iterations. Additional iterations were performed in the case that the effective sample size *N*_*eff*_ > 10⋅*n*_θ_ was not fulfilled. The Kish effective sample size was *N*_*eff*_ used (Beskos et al., 2014), which is defined as


(6) $$N_{e\,f\,f}=\frac{{\left[\sum_{\theta \in \Theta }w\,\left(\theta \right)\right]}^{2}}{\sum_{\theta \in \Theta }\left(w\,{\left(\theta \right)}^{2}\right)}.$$

### Problem Description

#### Clinical Data

Patient-specific simulations were based on echocardiographic data from AC mutation carriers in various disease stages. Besides clinically measured LV and RV regional deformation imaging data, the LV end-diastolic volume (EDV), LV ejection fraction (EF), and right ventricular basal diameter (RVD) were used as model input. We used echocardiographic data of nine pathogenic AC mutation carriers which were evaluated in the University Medical Center Utrecht, Netherlands. As previously described (van Osta et al., 2021), deformation analyses of these echocardiograms were performed twice by two observers to determine clinical inter- and intra-observer variability. Lastly, longitudinal datasets with >2 echocardiograms per patient at different time points were used to explore applicability of the model for follow-up of tissue properties over time. These longitudinal datasets were acquired from AC mutations carriers which were evaluated in the Oslo University Hospital, Norway.

All echocardiographic data were obtained on a Vivid 7, Vivid 9, or Vivid E95 ultrasound machine (GE Vingmed, Horten, Norway). The echocardiographic protocol was described previously (Kirkels et al., 2021). In this study, we focused on the right ventricular free wall (RVfw). This is typically the most affected area in AC mutation carriers (Marcus et al., 2010), which is expressed in typical deformation abnormalities (delayed onset of shortening, decreased peak systolic strain, post-systolic shortening, and increased RV mechanical dispersion) (Kirkels et al., 2021). Therefore, deformation patterns of three RVfw segments (apical, mid-ventricular, and basal) were used as input for our modeling framework (Figure 1) (van Osta et al., 2021). Additionally, LV free wall (LVfw) and interventricular septal (IVS) deformation patterns were included to ensure realistic mechanical boundary conditions for the RVfw in terms of ventricular interaction. These patterns were obtained by averaging the 12 LVfw and 6 IVS segmental deformation curves, respectively, using the standardized 18-segment model (Voigt et al., 2015).

#### Computational Model of Heart and Circulation

Clinical measures were simulated using the CircAdapt model. This model is a fast biomechanical lumped parameter model of the heart and circulation. *Via* the one fiber model (Arts et al., 2005), wall stress is related to cavity pressure. The TriSeg module allows inter-ventricular interaction over the IVS (Lumens et al., 2009). Phenomenological material laws prescribe the stress–strain relation in the spherical walls. The MultiPatch module allows for regional heterogeneity of tissue properties within a single wall (Walmsley et al., 2015) and is used to describe the heterogeneity in the RVfw. Three segments were created in the RVfw to model the mechanics in the three different RVfw segments (apical, mid-ventricular, and basal).

The parameter subset θ included for estimation was based on a previous sensitivity analysis (van Osta et al., 2021) and is shown in Table 1. Parameters included were regional parameters describing the constitutive behavior of active (SfAct) and passive stress (k1), activation delay (dT), reference wall area (AmRef), and global parameters relative systole duration (RSD), and CO. Heart rate (HR) in the model was set to match clinically measured HR to ensure equal cycle lengths in measured and modeled signals.

**TABLE 1:** parameters included in this study.

Model parameter	Unit	Description	Sample distribution	Parameter range	N parameters	
SfAct	kPa	Active stress scaling factor	logit-uniform	[0, 1000]	5	[LVfw, IVS, RVapex, RVmid, RVbase]
k1	–	Stiffness exponent	logit-uniform	[1, 100]	5	[LVfw, IVS, RVapex, RVmid, RVbase]
dT	ms	Activation delay	logit-uniform	[−200, 800]	5	[LVfw, IVS, RVapex, RVmid, RVbase]
AmRef	cm^2^	Eccentric hypertrophy	log-uniform	[0,∞]	3	[LVfw, IVS, RVfw]
RSD	–	Global systolic duration scaling	log-uniform	[0,∞]	1	Global
Q0	L/min	Cardiac Output	log-uniform	[0,∞]	1	Global
					20	

Strain was defined as the segmental displacement relative to its reference length at end diastole (see Supplementary Material 1.2). Additionally, EF, EDV, and RVD were included. Modeled EDV was defined as the maximum cavity volume of the LV cavity assuming perfect valve behavior. EF was defined as the ratio of stroke volume over maximum volume. RVD was defined as the maximum cavity diameter between the RVfw and IVS.

#### Likelihood Function

As shown in Equation 4, the likelihood function was based on the summed squared error X^2^. This error consists of the error in strain of the five segments and on the error in EF, EDV, and RVD. Because the measured diastolic strain is less reliable due to the drift affecting most of this phase, we only included strain during the systolic phase in this study. This systolic phase was defined from the onset of the QRS complex until 100 ms after peak strain of the segment with longest shortening phase.

To account for dependencies in strain, we included weighted dimensionless errors based on strain ($e_{\varepsilon ,s\,e\,g}^{2})$, strain rate ($e_{\bar{\varepsilon },s\,e\,g}^{2})$, and inter-segmental strain differences ($e_{\varepsilon_{i\,n\,t\,e\,r}}^{2}$). Errors in EF ($e_{E\,F}^{2}$), EDV ($e_{E\,D\,V}^{2}$), and RVD ($e_{R\,V\,D}^{2}$) were assumed independent, resulting in the X^2^ to be the sum of all individual weighted dimensionless errors *e*^2^:


(7) $${\text{X}}^{2}=\sum_{s\,e\,g\in s\,e\,g\,m\,e\,n\,t\,s}\left(e_{\varepsilon ,s\,e\,g}^{2}+e_{\bar{\varepsilon },s\,e\,g}^{2}\right)+\sum_{i\,n\,t\,e\,r\in i\,n\,t\,e\,r\,s\,e\,g}e_{\Delta \,\varepsilon_{i\,n\,t\,e\,r}}^{2}+\sum_{m\in \left[E\,F,E\,D\,V,R\,V\,D\right]}e_{m}^{2}.$$

Standard deviations used to normalize each individual term were manually estimated *a priori* to meet differences between the inter- and intraobserver datasets. Standard deviations used to normalize EF, EDV, and RVD were set *a priori* in consultation with clinical partners. A more detailed description of the likelihood function is included in Supplementary Material 1.2.

#### Right Ventricle Tissue Properties

To relate our simulations to clinical measures, four RV tissue properties were investigated, namely contractility, activation delay, compliance, and myocardial work. These measures are explained in more detail in Supplementary Material 1.5. In brief, segmental contractility was defined as the maximum rate of active stress rise, which can be seen as the equivalent of the maximum rate of ventricular systolic pressure rise (*dP*/*dt*_*max*_) on a local tissue level. Segmental wall compliance was defined as the slope of the end-diastolic myofibre stress–strain relationship at time before first ventricular activation and can be interpreted as the regional equivalent of the slope of the global end-diastolic pressure–volume relation. Myocardial work density was defined as the area within the stress–strain loop and can be interpreted as the regional equivalent of global stroke work.

### Simulation Protocol

#### Uncertainty Quantification of Real Patient Datasets

Nine clinical datasets in which the echocardiographic images were analyzed twice by two independent observers were included to test reproducibility, leading to 36 datasets. For each individual dataset, parameters were estimated three times resulting in 108 estimations in total. Since no ground truth exists for estimated model parameters, only the reproducibility of estimations was evaluated. Three kinds of reproducibility were investigated, namely computational reproducibility, reproducibility including interobserver variability, and reproducibility including intraobserver variability. First, computational reproducibility was defined as the reproducibility of the exact same clinical dataset and quantified by the mutual information (MI) between two model parameter estimations. The same protocol was repeated three times with a different random seed. To calculate the MI, two distributions were discretized into 100 bins. The MI was then defined as the overlap divided by the union of the distributions. Secondly, reproducibility including interobserver variability was tested on the nine patient datasets, whereby a second blinded observer performed deformation imaging analysis on the same echocardiographic loops as the first observer. It was defined as MI between two estimated model parameter distributions from two datasets observed by the two different observers. Finally, reproducibility including intraobserver variability was quantified similarly from two different datasets, whereby the observer performed the deformation analysis again after at least 2 weeks, blinded to previous results. The median MI with 95% confidence interval (CI) of all parameter estimations was reported. In case the estimations from different observations fully overlap, MI = 100%. In case of no overlap at all, MI = 0%.

#### Uncertainty Quantification of Virtual Patient Datasets

To test the trueness of the estimation, *in silico* generated virtual patients were generated. To ensure these virtual patients to be representable for real AC patients, nine virtual patients were created based on the nine real patient datasets. For each real patient, the simulation with maximum likelihood was selected. The output of this simulation was used as virtual patient dataset, which was used as input of the modeling framework.

Trueness of the virtual estimations was tested by comparing the estimated distribution with the known true parameter values. For each parameter, the highest density interval (HDI) for which the true value is in the interval was calculated. The HDI was defined as the area of the distribution for which the posterior holds *p*(θ|*z*) = *p*(θ_*true*_|*z*). The distribution was approximated with a histogram with bin width defined by the Freedman–Diaconis rule (Freedman and Diaconis, 1981). The HDI for each parameter should be near 0% meaning the true value is near the maximum *a posteriori*.

#### Application in Longitudinal Datasets

Two subjects with a baseline and two follow-up echocardiograms were selected (Table 2). For all six datasets, clinical data was extracted and the datasets were estimated independently of each other, similarly as described above. The two longitudinal sets of estimated tissue properties were investigated. Due to the retrospective nature of this study, LV EDV was only available at baseline. We assumed that it did not change during follow-up.

**TABLE 2:** Patient characteristics of the two subjects at baseline and follow-up used in the likelihood function.

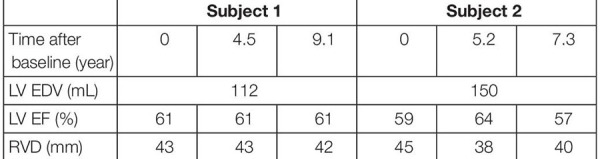

### Code Implementation

The CircAdapt model was written in C++. All other code was written in Python. Each individual dataset was solved sequentially and independently. The source code of the CircAdapt model has been made available before (van Osta et al., 2020). All other source code is publicly available on Zenodo^1^. Datasets were estimated in parallel with Python 3.9.4 on a AMD Ryzen Threadripper 3970X.

## Results

### Uncertainty Quantification of Real Patient Datasets

**FIGURE 3:**
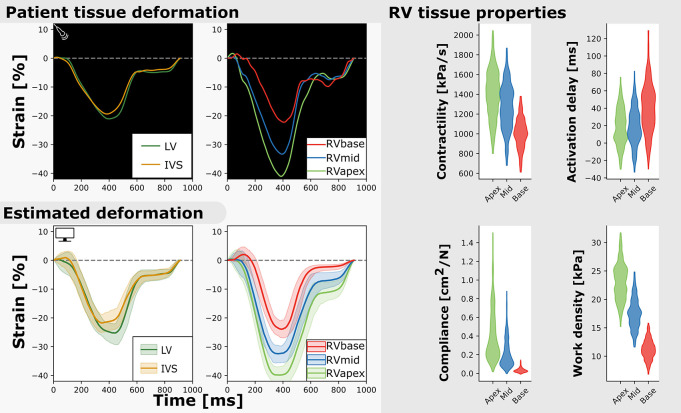
Measured and estimated strain of real subject (left) and violin plots of estimated parameters (right). Deformation patterns and regional heterogeneity was well captured by the model. The best simulation in the sample set was in good agreement with to the patients dataset (**${\text{X}}_{o\,p\,t}^{2}=8.9)$**.

**FIGURE 4:**
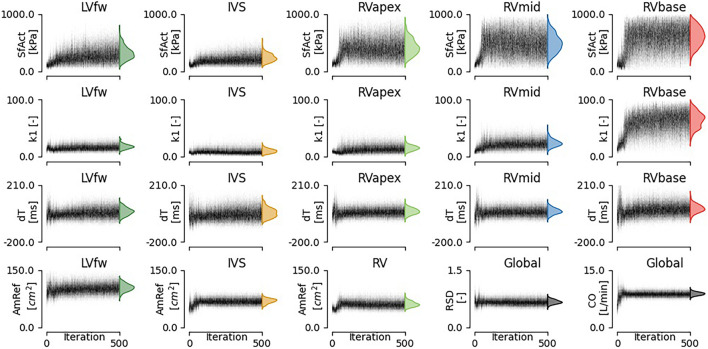
Convergence of estimated model parameters. The distributions on the right show the final estimated posterior distribution.

Regional deformation characteristics were accurately simulated close to the measured deformation and with reasonable uncertainty {${\text{X}}_{o\,p\,t}^{2}=9.4\left(95\%CI\left[5.4-20.9\right]\right)\}$. Figure 3 (left) shows a representative example. The modeled strain followed the pattern of clinically measured strain during systole and heterogeneity between the segments was well captured. A 1D representation of the convergence of the proposal distribution, corresponding to the estimated model parameters is shown in Figure 4. In the first 50 iterations, the proposal distribution decreased, increased, and moved to the area of interest. From the 50th iteration, most proposal distributions stabilized. This behavior was also observed in estimations in other datasets (see Supplementary Material 2.2).

**FIGURE 5:**
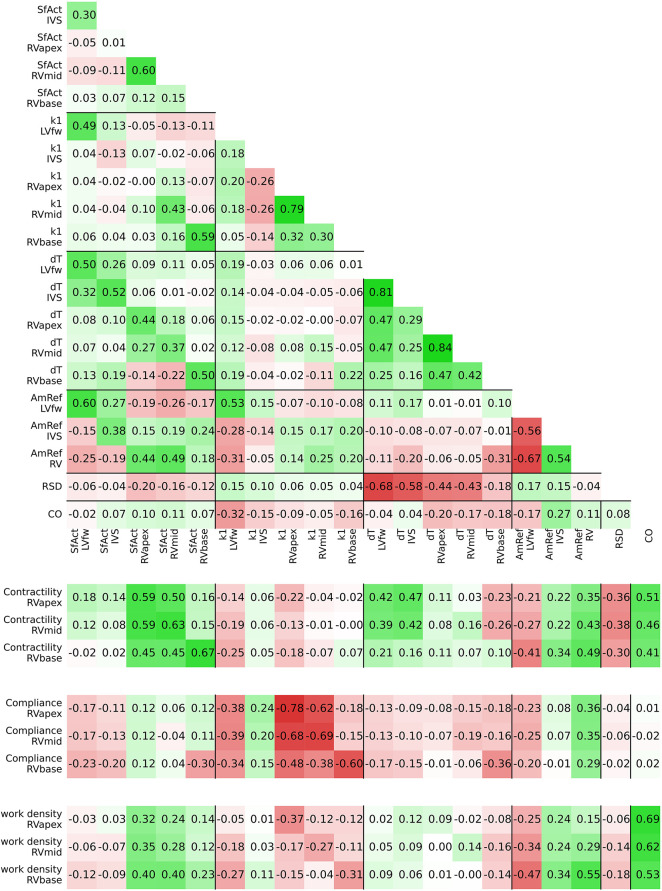
Posterior correlation matrix of the estimated model parameters (top) and the correlation between the posterior distribution of model parameters and derived tissue properties (bottom).

The estimated posterior distributions of the model parameters (Figure 4) of most parameters were estimated with small variances, except for parameters SfAct and k1, because they were unidentifiable in some segments. The posterior correlation matrix (Figure 5, top) shows the correlation between estimated posterior distributions. Notable are the correlations between model parameters SfAct, k1, dT, and AmRef describing mechanics in the same wall segment. Additionally, there was a high correlation between different segments for the model parameters dT and AmRef. From the two global parameters, only RSD seemed to correlate with dT.

Figure 3 (right) shows the estimated regional RV model parameters and the RV tissue properties contractility, activation delay, compliance, and work density. The RV tissue properties were estimated with distributions with a smaller variance compared to the estimated model parameters. A decrease in basal contractility, compliance, and work density with respect to the apical and mid segment was found which is in line with the abnormal basal deformation pattern.

Figure 5 (bottom) shows the correlation between posterior model parameter distributions with the RV tissue properties contractility, compliance, and work density. Contractility was mostly correlated with SfAct, AmRef, and CO. In the RVapex and RVmid, contractility was not only dependent on the parameters prescribing its own segmental mechanics, but also on the parameters prescribing other segmental mechanics. Similar results were observed for compliance, which was correlated with SfAct, k1, and dT. Compliance showed no correlation with AmRef, RSD, and CO. Work density was mostly correlated with CO.

Estimated model parameters were highly reproducible. Computational reproducibility was found with an MI of 89.9% (95% CI [60.1–95.9]). The reproducibility error given inter- and intraobserver variability were estimated with an MI of 86.5% (95% CI [46.0–95.2]) and 85.9% (95% CI [43.7–95.3]), respectively. More details on reproducibility and inter- and intraobserver variability are shown in Supplementary Material 2.1.

### Uncertainty Quantification of Virtual Patient Datasets

**FIGURE 6:**
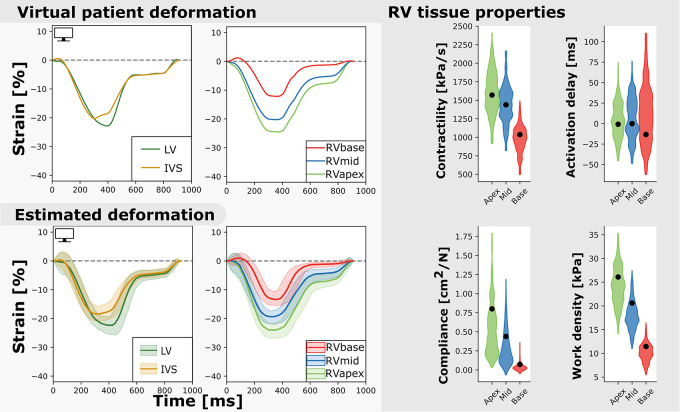
Measured and estimated strain of virtual subject (left) and violin plots of estimated parameters (right). The estimated properties are close to the true properties of the virtual patient (black dot) and the heterogeneity is well captured. The best simulation in the sample set was closely related to the virtual patients dataset (**${\text{X}}_{o\,p\,t}^{2}=2.0)$**.

Nine virtual patients were created based on the nine real-patient estimations. As an example, Figure 6 shows the virtual patient based on the patient results described above. Regional deformation characteristics were simulated close to the virtual patients deformation characteristics (${\text{X}}_{o\,p\,t}^{2}=2.0\left(95\%CI=\left[1.2-3.0\right]\right)$. The true parameter values were well captured by the estimated distributions. The HDI of the true parameter values was 9% (95% CI [0–79]). Heterogeneity in model parameters was well preserved. The width of the distribution in virtual fits was similar to that in the original patient estimation.

### Application: Longitudinal Datasets

**FIGURE 7:**
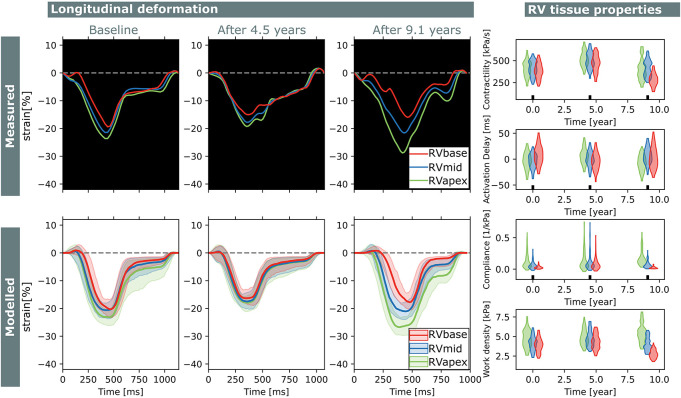
Longitudinal estimations subject 1. Echocardiographic deformation imaging was performed at baseline, and after 4.5 and 9.1 years of follow-up. Computer simulations showed homogeneous RV contractility, activation delay, compliance, and work at baseline. At last follow-up, subject 1 developed an abnormal deformation pattern of the basal RV. Estimation of RV tissue properties from these deformation data showed an apex-to-base heterogeneity in activation delay, compliance, and work density.

**FIGURE 8:**
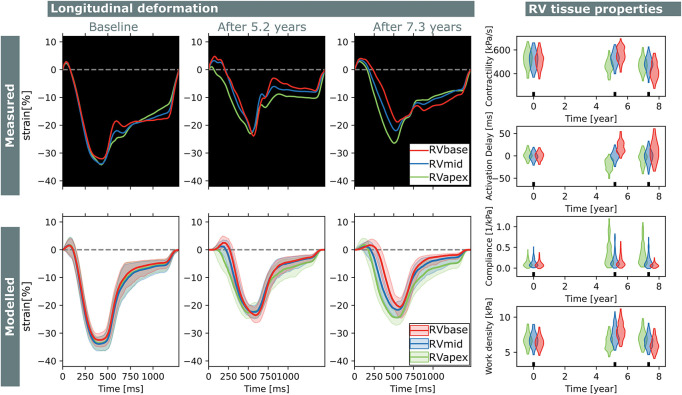
Longitudinal estimations subject 2. Echocardiographic deformation imaging was performed at baseline, and after 5.2 and 7.3 years of follow-up. Subject 2 had normal RV deformation patterns at baseline and did not develop clear deformation abnormalities during follow-up. Contractility, compliance, and work density were estimated homogeneously at baseline.

Two subjects with a baseline and two follow-up echocardiograms were included in this study (Table 2). The first subject had a follow-up examination after 4.5 and 9.1 years and the second subject after 5.2 and 7.3 years. Results of these case studies are shown in Figures 7, 8.

Subject 1 developed an abnormal deformation pattern of the basal RV segment at last follow-up which was not seen at baseline. Computer simulations showed homogeneous RV contractility, activation delay, compliance, and work at baseline. In the last follow-up examination, an apex-to-base heterogeneity in compliance and work density was present.

Subject 2 showed normal RV deformation patterns at baseline and did not develop clear deformation abnormalities during follow-up. Contractility, activation delay, compliance, and work density were estimated homogeneously at baseline. In the final follow-up, a small apex-to-base heterogeneity in compliance was present.

## Discussion

In this work, we successfully applied AMIS to estimate posterior distributions of model parameters describing local passive and active tissue behavior based on echocardiographic deformation measurements. Estimated deformation closely resembled the clinically measured myocardial deformation with a realistic level of uncertainty originating from both the measurement and the model. Estimated RV tissue properties reflected progression of the disease substrate over time present in the clinical case studies.

### Model-Based Inference

Personalization of cardiac computational models is becoming more popular and several approaches have been proposed. Schiavazzi et al. (2017) used MCMC to estimate model parameters in a simplified model of the single-ventricular heart in a close-looped circulation, based on clinically measured pressures and flows. Corrado et al. (2015) used a Reduced Order Unscented Kalman Filter to estimate model parameters to optimize body surface potential maps and myocardial displacement. Meiburg et al. (2021) used the Unscented Kalman Filter to predict post-intervention hemodynamics after trans-aortic valve implantation. Zenker (2010) used importance sampling to estimate model parameters in a cardiovascular model. Dhamala et al. (2020) used high-dimensional Bayesian optimization for parameter personalization of a cardiac electrophysiological model. Coveney and Clayton (2018) used history matching to calibrate the maximum conductance of ion channels and exchangers in two detailed models of the human atrial action potential against measurements of action potential biomarkers. Daly et al. (2017) used sequential Monte Carlo Approximate Bayesian Inference to quantify the uncertainty amplification resulting in a cellular action potential model. Camps et al. (2021) used the same technique to estimate key ventricular activation properties based on non-invasive electrocardiography and cardiac magnetic resonance imaging.

These studies used computational models with different levels of model complexity in both anatomical and physiological detail. Complex models allow personalization with a high number of details, however, they suffer from a high-dimensional unknown space increasing the difficulty of personalization due to unidentifiability of the model parameters. This problem can be solved by reducing the complexity of the optimization problem by assuming global model parameters (Davies et al., 2019) or regional model parameters (Dhamala et al., 2017). However, this does not reduce the computational cost and increases model discrepancy. It is suggested to use a surrogate model to approximate the exact posterior probability density function (Paun et al., 2019), but this creates a new source of uncertainty. Including model discrepancy in the estimation often fails due to the non-identifiability between model parameter estimations and model discrepancy (Lei et al., 2020). The pseudo-true parameter value found by ignoring model discrepancy can still be valuable for clinical interpretation.

Another approach is to reduce the complexity of the model. Various lumped parameter models of the heart and circulation have been used for fast personalization (Zenker, 2010; Schiavazzi et al., 2017; Meiburg et al., 2021). The cost of low complexity may lead to an increase in model discrepancy due to model assumptions and simplifications (Lei et al., 2020). It was, however, demonstrated before that the CircAdapt model is highly efficient in simulating regional mechanics and is able to simulate realistic hemodynamics (Arts et al., 2012; Walmsley et al., 2015). We previously showed that the CircAdapt model can simulate segmental mechanics with a similar spatial resolution as in clinical strain imaging measurements with low discrepancy (Walmsley et al., 2015; van Osta et al., 2021). Therefore, we assume the CircAdapt model is a suitable model for modeling regional strain in AC patients.

In this study, we chose importance sampling because it is highly effective for complex high-dimensional models (Bugallo et al., 2017). The computational cost of our model was approximately 1000 times higher compared to the calculation of the probability density of a sample drawn from the proposal distribution. Therefore, AMIS was the most suitable variant to optimally reuse all samples (Cornuet et al., 2012).

Efficiency of AMIS heavily depends on the definition of the proposal distribution (Bugallo et al., 2017). A wider proposal distribution ensures to visit the full input space of interest, but is accompanied by a risk of non-converging estimations due to the high number of samples with a low sample weight. On the other hand, a more narrowed search has the risk of finding a local minimum in which the wrong posterior is estimated, or the risk of collapsing when the weight of the found minimum drops to zero. As the number of samples goes to infinity, the sample weight will be equally distributed. However, for the limited number of samples drawn, an optimal balance should be found. We successfully implemented annealed adaptive importance sampling to prevent the model from premature convergence while still being able to narrow the proposal distribution in the later iterations. More research should go into defining the proposal distribution or the initial proposal distribution.

In this study, it took approximately 16 h per dataset to converge. This time includes generating the proposal distributions, generating samples, running simulations, obtaining the likelihood function, and calculating the sample weights. The total duration mainly depends on the duration of each individual simulation, since the number of iterations in the estimations was equal or close to 500. The duration of each simulation depended on HR, numerical stability, and number of beats needed to get a hemodynamically stable solution. Computational time can be reduced in future studies, since AMIS allows parallel calculation of simulations. This reduction in computational time will be essential for clinical application of our method on a larger scale.

### Uncertainty Quantification in Arrhythmogenic Cardiomyopathy

Cardiovascular models are, in general, complex models with a multitude of parameters. To create digital twins with the CircAdapt model, we used a parameter subset that we determined in a previous study (van Osta et al., 2020). This subset includes model parameters related to regional RV contractile function, compliance, and activation delay. This is in line with functional and structural myocardial changes found in AC patients [e.g., fibro-fatty replacement of myocytes (Basso et al., 2009), altered calcium handling (van Opbergen et al., 2019), and fibrosis (Tandri et al., 2005)] and early generic simulation based hypotheses (Mast et al., 2016). These structural changes might cause abnormal electrical activation observed in patients with AC (Haqqani et al., 2012). The RV tissue properties are useful to quantify the substrate, however, the model cannot distinguish the cellular origin of the substrate.

The likelihood function was based on our prior knowledge of the pathology. It is not trivial how to include this information as the amount of uncertainty and its dependencies is not known but heavily affects the posterior distribution. In this study, we limited the objectives in the likelihood function to only that information in the longitudinal study that our model can simulate realistically. The main contributor is regional RV strain, as regional deformation abnormalities are found in early stages of the disease (Sarvari et al., 2011; Mast et al., 2016, 2019; Leren et al., 2017; Lie et al., 2018; Malik et al., 2020). LV strain, RVD, LV EF, and LV EDV are included in the likelihood to personalize geometric properties of the model. Because of the complex geometry of the thin-walled RV, our 2D imaging methods did not provide a comprehensive measure of RV size and wall thickness. In future studies, 3D imaging methods might provide a more comprehensive inclusion of geometric variability of the RV. The RVD was included to account for large geometrical differences between patients and geometrical changes over time. Wall volumes were not included in the parameter subset because they were unidentifiable given the available measurements.

Dependencies in strain were partially included by including strain rate and strain differences. Based on the used likelihood function, posterior distributions were estimated with a relatively wide variance (Figure 4), suggesting not all parameters are identifiable. The low reproducibility in some parameters (HDI 95% CI [0–79%]) is probably related to this unidentifiability. Heterogeneity in model parameters is, however, well preserved, suggesting that measurements that are sensitive to segment-averaged model parameters should also be included in the likelihood function. Further prospective studies could investigate the error propagation of dependent and independent uncertainties, whether all components of the likelihood are essential to include, and which other measurements should be included to increase the identifiability of the model parameters.

Derived tissue properties were estimated more precise and reproducible compared to model parameters, suggesting that different parameter combinations can result in the same hemodynamic state. Mechanics of the three RV segments were modeled with the same mathematical equations, however, they have different interactions with the surrounding walls as shown in Figure 5. Compliance in the basal segment was estimated more precise compared to the other segments (Figure 6). This results from the non-linear behavior of the model, as basal model parameters were differently estimated due to basal deformation abnormalities. Therefore, compliance in the basal segment was less correlated with the other segments.

In this study, we used a single definition for myocardial contractility and compliance related to other more global definitions. There is no consensus on a single indicator for contractility and compliance, and often multiple (non-invasive) measures are used to get an impression. For contractility, the maximum pressure–time derivative *dP*/*dt*_*max*_ is the most commonly used index of contractility in the field of drug safety assessment (Sarazan et al., 2011). Although this measure is preload and afterload dependent, the regional stress–time derivative as local equivalent gives insight in the regional differences in RV contractile function. Other global measures have been proposed to bypass preload and afterload dependencies, such as *dP*/*dt*_*max*_ at a specific pressure (Sarazan et al., 2011) or end-systolic pressure–volume relation (Suga and Sagawa, 1974). New techniques might be useful for future validation of RV tissue properties, such as shear wave imaging (Pernot et al., 2011) to quantify cardiac stiffness.

The gold-standard assessment of RV stiffness (inverse of compliance) is the end-diastolic pressure–volume relation (El Hajj et al., 2020). The local equivalent is the models material law describing the stress–sarcomere length relation. The actual amount of stress prescribed by this law depends on the sarcomere length during the cycle (Arts et al., 2005). Due to the complexity of the model, which includes mechanics based on sarcomere length, an accurate estimation of compliance is difficult. The compliance measure as used in this study only includes the compliance at the end diastolic sarcomere length and is therefore load-dependent. To obtain a load-independent measure, more information on the loading conditions should be included in the likelihood distribution.

### Case Study and Future Research Directions

The two subjects included in the case study showed different behavior over time. The first subject developed an abnormal basal RV deformation pattern during follow-up which was reflected in changes in estimated local tissue properties. The second subject did not develop clear deformation abnormalities, but did develop slight abnormal heterogeneity in tissue properties. In both cases, only small changes in estimations were observed from baseline to follow-up. It has previously been shown that heterogeneity in deformation patterns has prognostic value for disease progression (Mast et al., 2019) and life-threatening arrhythmia (Sarvari et al., 2011). Although no further follow-up of these subjects was available, we can hypothesize our model might identify abnormal tissue substrates before this is clearly visible in deformation patterns. Further studies should investigate whether our approach is able to detect AC in an early stage and whether it has added prognostic value.

In this study, we estimated model parameters to predict tissue mechanics under mechanical loading similar to loading during measurement. To achieve this, we included CO in the parameter subset and EDV and EF in the likelihood function. The model could be used for predicting the behavior of the heart under different loading conditions. This could facilitate the study of loading effects of drug interventions in the digital twin. Besides, the effect of exercise, which is an important modulator of phenotypic expression of AC (Prior and La Gerche, 2020), could be studied in the digital twin. For the latter, a virtual cardiac exercise performance test as proposed by van Loon et al. (2020) could be used to give more insight in the severity of the substrate and possible triggers for disease progressions. To allow the CircAdapt model to extrapolate its state to other loading conditions such as exercise, more information should be included.

### Limitations

Uncertainties are assumed statistically independent and additive, however, this is in fact more complicated. Measurements have multiple sources for uncertainty. We have only included inter- and intra-observer variability of the speckle tracking imaging in our study. Global longitudinal strain has proven to be reproducible, however, it has been shown that beat-to-beat variability affects segmental peak strain, end systolic strain and post-systolic strain (Mirea et al., 2018). More research should elucidate the origin of this uncertainty, its effect on normalized strain morphology as included in our study, and how to optimally include uncertainty in defining the likelihood function. This could also facilitate inclusion of realistic noise on virtual patient datasets, which was outside the scope of this study.

Arrhythmogenic cardiomyopathy is not only characterized by structural disease manifestation, but electrophysiologic substrates play an important role as well (Groeneweg et al., 2015). Currently, the CircAdapt model only contains the lumped effect of electrophysiology to describe the mechanical behavior. Future studies could extend the model with a more detailed electromechanical coupling, such as proposed by Lyon et al. (2020), to be able to describe the electrophysiologic substrate.

## Conclusion

We presented a patient-specific modeling approach taking into account uncertainties. With this approach, we were able to reproduce regional ventricular deformation patterns and estimate the underlying tissue properties in AC mutation carriers with an acceptable level of uncertainty. Virtual estimations were precise and real-world estimations were highly reproducible. Two subjects in our case study revealed the evolution of early-stage AC disease over time using longitudinal follow-up datasets. Future studies should apply our method on a larger cohort and investigate the course of early stage RV disease development at individual as well as patient population levels.

## Data Availability Statement

The source code of the CircAdapt model has been made available before (van Osta et al., 2020). The binaries of the CircAdapt model, all other source code, and the virtual patient data generated for this study can be found on Zenodo (https://doi.org/10.5281/zenodo.5084657).

## Ethics Statement

The studies involving human participants were reviewed and approved by the local institutional ethics review boards. Written informed consent was obtained in accordance with the Declaration of Helsinki.

## Author Contributions

NO and FK contributed to conception and design of the study and wrote the manuscript. NO performed the simulations. FK provided the clinical data. TL, TK, AL, RM, WH, MC, TD, KH, AT, and JL helped with analysis and interpretation of the data. All co-authors critically read the manuscript and approved it. All authors contributed to the article and approved the submitted version.

## Conflict of Interest

The authors declare that the research was conducted in the absence of any commercial or financial relationships that could be construed as a potential conflict of interest.

## Publisher’s Note

All claims expressed in this article are solely those of the authors and do not necessarily represent those of their affiliated organizations, or those of the publisher, the editors and the reviewers. Any product that may be evaluated in this article, or claim that may be made by its manufacturer, is not guaranteed or endorsed by the publisher.

## Footnotes

1
https://doi.org/10.5281/zenodo.5084657

## References

[B1] Arts T. Delhaas T. Bovendeerd P. Verbeek X. Prinzen F. (2005). Adaptation to mechanical load determines shape and properties of heart and circulation: the CircAdapt model. *Am. J. Physiol. Heart Circ. Physiol.* 288 1943–1954. 10.1152/ajpheart.00444.2004 15550528

[B2] Arts T. Lumens J. Kroon W. Delhaas T. (2012). Control of whole heart geometry by intramyocardial mechano-feedback: a model study. *PLoS Comput. Biol.* 8:e1002369. 10.1371/journal.pcbi.1002369 22346742 PMC3276542

[B3] Basso C. Corrado D. Marcus F. I. Nava A. Thiene G. (2009). Arrhythmogenic right ventricular cardiomyopathy. *Lancet* 373 1289–1300. 10.1016/S0140-6736(09)60256-7 19362677

[B4] Beskos A. Crisan D. Jasra A. (2014). On the stability of sequential Monte Carlo methods in high dimensions. *Ann. Appl. Probab.* 24 1396–1445. 10.1214/13-AAP951

[B5] Bugallo M. F. Elvira V. Martino L. Luengo D. Miguez J. Djuric P. M. (2017). Adaptive importance sampling: the past, the present, and the future. *IEEE Signal Process. Mag.* 34 60–79. 10.1109/MSP.2017.2699226

[B6] Camps J. Lawson B. Drovandi C. Minchole A. Wang Z. J. Grau V. (2021). Inference of ventricular activation properties from non-invasive electrocardiography. *Med. Image Anal.* 73:102143. 10.1016/j.media.2021.102143 34271532 PMC8505755

[B7] Černý V. (1985). Thermodynamical approach to the traveling salesman problem: an efficient simulation algorithm. *J. Optim. Theory. Appl.* 45, 41–51. 10.1007/BF00940812

[B8] Cornuet J. M. Marin J. M. Mira A. Robert C. P. (2012). Adaptive multiple importance sampling. *Scand. J. Stat.* 39 798–812. 10.1111/j.1467-9469.2011.00756.x

[B9] Corrado C. Gerbeau J. F. Moireau P. (2015). Identification of weakly coupled multiphysics problems. Application to the inverse problem of electrocardiography. *J. Comput. Phys.* 283 271–298. 10.1016/j.jcp.2014.11.041

[B10] Corral-Acero J. Margara F. Marciniak M. Rodero C. Loncaric F. Feng Y. (2020). The ‘Digital Twin’ to enable the vision of precision cardiology. *Eur. Heart J.* 41 4556–4564. 10.1093/eurheartj/ehaa159 32128588 PMC7774470

[B11] Coveney S. Clayton R. H. (2018). Fitting two human atrial cell models to experimental data using Bayesian history matching. *Prog. Biophys. Mol. Biol.* 139 43–58. 10.1016/j.pbiomolbio.2018.08.001 30145156

[B12] Daly A. C. Cooper J. Gavaghan D. J. Holmes C. (2017). Comparing two sequential Monte Carlo samplers for exact and approximate Bayesian inference on biological models. *J. R. Soc. Interface* 14:20170340. 10.1098/rsif.2017.0340 28931636 PMC5636270

[B13] Davies V. Noè U. Lazarus A. Gao H. Macdonald B. Berry C. (2019). Fast parameter inference in a biomechanical model of the left ventricle by using statistical emulation. *J. R. Stat. Soc. Ser. C Appl. Stat.* 68 1555–1576. 10.1111/rssc.12374 31762497 PMC6856984

[B14] Dhamala J. Arevalo H. J. Sapp J. Horácek B. M. Wu K. C. Trayanova N. A. (2018). Quantifying the uncertainty in model parameters using Gaussian process-based Markov chain Monte Carlo in cardiac electrophysiology. *Med. Image Anal.* 48 43–57. 10.1016/j.media.2018.05.007 29843078 PMC6076346

[B15] Dhamala J. Arevalo H. J. Sapp J. Horacek M. Wu K. C. Trayanova N. A. (2017). Spatially adaptive multi-scale optimization for local parameter estimation in cardiac electrophysiology. *IEEE Trans. Med. Imaging* 36 1966–1978. 10.1109/TMI.2017.2697820 28459685 PMC5687096

[B16] Dhamala J. Bajracharya P. Arevalo H. J. Sapp J. L. L. Horácek B. M. Wu K. C. (2020). Embedding high-dimensional Bayesian optimization via generative modeling: parameter personalization of cardiac electrophysiological models. *Med. Image Anal.* 62:101670. 10.1016/j.media.2020.101670 32171168 PMC7237332

[B17] El Hajj M. C. Viray M. C. Tedford R. J. (2020). Right heart failure: a hemodynamic review. *Cardiol. Clin.* 38 161–173. 10.1016/j.ccl.2020.01.001 32284094

[B18] Freedman D. Diaconis P. (1981). On the histogram as a density estimator:L2 theory. *Z. Wahrscheinlichkeitstheorie Verw. Gebiete* 57 453–476. 10.1007/BF01025868

[B19] Groeneweg J. A. Bhonsale A. James C. A. te Riele A. S. Dooijes D. Tichnell C. (2015). Clinical presentation, long-term follow-up, and outcomes of 1001 arrhythmogenic right ventricular dysplasia/cardiomyopathy patients and family members. *Circ. Cardiovasc. Genet.* 8 437–446. 10.1161/CIRCGENETICS.114.001003 25820315

[B20] Haqqani H. M. Tschabrunn C. M. Betensky B. P. Lavi N. Tzou W. S. Zado E. S. (2012). Layered activation of epicardial scar in arrhythmogenic right ventricular dysplasia possible substrate for confined epicardial circuits. *Circ. Arrhythm. Electrophysiol.* 5 796–803. 10.1161/CIRCEP.111.967935 22634228

[B21] Kirkels F. P. Lie Ø. H. Cramer M. J. Chivulescu M. Rootwelt-Norberg C. Asselbergs F. W. (2021). Right ventricular functional abnormalities in arrhythmogenic cardiomyopathy: association with life-threatening ventricular arrhythmias. *JACC Cardiovasc. Imaging* 14 900–910. 10.1016/j.jcmg.2020.12.028 33582062

[B22] Lei C. L. Ghosh S. Whittaker D. G. Aboelkassem Y. Beattie A. Cantwell C. D. (2020). Considering discrepancy when calibrating a mechanistic electrophysiology model subject areas. *Philos. Trans. A Math. Phys. Eng. Sci.* 378:20190349. 10.1098/rsta.2019.0349 32448065 PMC7287333

[B23] Leren I. S. Saberniak J. Haland T. F. Edvardsen T. Haugaa K. H. (2017). Combination of ECG and echocardiography for identification of arrhythmic events in early ARVC. *JACC Cardiovasc. Imaging* 10 503–513. 10.1016/j.jcmg.2016.06.011 27771401

[B24] Li W. Lin G. (2015). An adaptive importance sampling algorithm for Bayesian inversion with multimodal distributions. *J. Comput. Phys.* 294, 173–190. 10.1016/j.jcp.2015.03.047

[B25] Lie Ø. H. Rootwelt-Norberg C. Dejgaard L. A. Leren I. S. Stokke M. K. Edvardsen T. (2018). Prediction of life-threatening ventricular arrhythmia in patients with arrhythmogenic cardiomyopathy. *JACC Cardiovasc. Imaging* 11 1377–1386. 10.1016/j.jcmg.2018.05.017 30031702

[B26] Lumens J. Delhaas T. Kirn B. Arts T. (2009). Three-wall segment (TriSeg) model describing mechanics and hemodynamics of ventricular interaction. *Ann. Biomed. Eng.* 37 2234–2255. 10.1007/s10439-009-9774-2 19718527 PMC2758607

[B27] Lyon A. Dupuis L. J. Arts T. Crijns H. J. G. M. Prinzen F. W. Delhaas T. (2020). Differentiating the effects of β-adrenergic stimulation and stretch on calcium and force dynamics using a novel electromechanical cardiomyocyte model. *Am. J. Physiol. Hear Circ. Physiol.* 319 H519–H530. 10.1152/ajpheart.00275.2020 32734816

[B28] Malik N. Win S. James C. A. Kutty S. Mukherjee M. Gilotra N. A. (2020). Right ventricular strain predicts structural disease progression in patients with arrhythmogenic right ventricular cardiomyopathy. *J. Am. Heart Assoc.* 9:e015016. 10.1161/JAHA.119.015016 32242475 PMC7428652

[B29] Marcus F. I. McKenna W. J. Sherrill D. Basso C. Bauce B. Bluemke D. A. (2010). Diagnosis of arrhythmogenic right ventricular cardiomyopathy/dysplasia: proposed modification of the task force criteria. *Eur. Heart J.* 31 806–814. 10.1093/eurheartj/ehq025 20172912 PMC2848326

[B30] Mast T. P. Taha K. Cramer M. J. Lumens J. van der Heijden J. F. Bouma B. J. (2019). The prognostic value of right ventricular deformation imaging in early arrhythmogenic right ventricular cardiomyopathy. *JACC Cardiovasc. Imaging* 12 446–455. 10.1016/j.jcmg.2018.01.012 29550307

[B31] Mast T. P. Teske A. J. Walmsley J. van der Heijden J. F. van Es R. Prinzen F. W. (2016). Right ventricular imaging and computer simulation for electromechanical substrate characterization in arrhythmogenic right ventricular cardiomyopathy. *J. Am. Coll. Cardiol.* 68 2185–2197. 10.1016/j.jacc.2016.08.061 27855808

[B32] Meiburg R. Zelis J. M. van ’t Veer J. M. van Velthoven S. J. A. van de Vosse F. N. Tonino P. A. L. (2021). Model-based aortic power transfer: a potential measure for quantifying aortic stenosis severity based on measured data. *Med. Eng. Phys.* 90 66–81. 10.1016/j.medengphy.2021.02.009 33781481

[B33] Mirea O. Pagourelias E. D. Duchenne J. Bogaert J. Thomas J. D. Badano L. P. (2018). Variability and reproducibility of segmental longitudinal strain measurement: a report from the EACVI-ASE strain standardization task force. *JACC Cardiovasc. Imaging* 11 15–24. 10.1016/j.jcmg.2017.01.027 28528147

[B34] Neal R. M. (2001). Annealed importance sampling. *Stat. Comput.* 11, 125–139. 10.1023/A:1008923215028

[B35] Niederer S. A. Lumens J. Trayanova N. A. (2019). Computational models in cardiology. *Nat. Rev. Cardiol.* 16 100–111. 10.1038/s41569-018-0104-y 30361497 PMC6556062

[B36] Paun L. M. Colebank M. Qureshi U. Olufsen M. Hill N. Husmeier D. (2019). “MCMC with delayed acceptance using a surrogate model with an application to cardiovascular fluid dynamics,” in *Proceedings of the International Conference on Statistics: Theory and Applications (ICSTA’19)*, Lisbon, 1–8. 10.11159/icsta19.28

[B37] Pernot M. Couade M. Mateo P. Crozatier B. Fischmeister R. Tanter M. (2011). Real-time assessment of myocardial contractility using shear wave imaging. *J. Am. Coll. Cardiol.* 58 65–72. 10.1016/j.jacc.2011.02.042 21700091

[B38] Prior D. La Gerche A. (2020). Exercise and arrhythmogenic right ventricular cardiomyopathy. *Heart Lung Circ.* 29 547–555. 10.1016/j.hlc.2019.12.007 31964580

[B39] Sarazan R. D. Mittelstadt S. Guth B. Koerner J. Zhang J. Pettit S. (2011). Cardiovascular function in nonclinical drug safety assessment: current issues and opportunities. *Int. J. Toxicol.* 30 272–286. 10.1177/1091581811398963 21527643

[B40] Sarvari S. I. Haugaa K. H. Anfinsen O. G. Leren T. P. Smiseth O. A. Kongsgaard E. (2011). Right ventricular mechanical dispersion is related to malignant arrhythmias: a study of patients with arrhythmogenic right ventricular cardiomyopathy and subclinical right ventricular dysfunction. *Eur. Heart J.* 32 1089–1096. 10.1093/eurheartj/ehr069 21406439

[B41] Schiavazzi D. E. Baretta A. Pennati G. Hsia T. Y. Marsden A. L. (2017). Patient-specific parameter estimation in single-ventricle lumped circulation models under uncertainty. *Int. J. Numer. Method Biomed. Eng.* 33:10. 10.1002/cnm.2799 27155892 PMC5499984

[B42] Suga H. Sagawa K. (1974). Instantaneous pressure volume relationships and their ratio in the excised, supported canine left ventricle. *Circ. Res.* 35 117–126. 10.1161/01.RES.35.1.117 4841253

[B43] Tandri H. Saranathan M. Rodriguez E. R. Martinez C. Bomma C. Nasir K. (2005). Noninvasive detection of myocardial fibrosis in arrhythmogenic right ventricular cardiomyopathy using delayed-enhancement magnetic resonance imaging. *J. Am. Coll. Cardiol.* 45 98–103. 10.1016/j.jacc.2004.09.053 15629382

[B44] Thiene G. Nava A. Corrado D. Rossi L. Pennelli N. (1988). Right ventricular cardiomyopathy and sudden death in young people. *N. Engl. J. Med.* 318 129–133. 10.1056/NEJM198801213180301 3336399

[B45] van Loon T. Knackstedt C. Cornelussen R. Reesink K. D. Brunner La Rocca H.-P. Delhaas T. (2020). Increased myocardial stiffness more than impaired relaxation function limits cardiac performance during exercise in heart failure with preserved ejection fraction: a virtual patient study. *Eur. Heart J. Digit. Health* 1 40–50. 10.1093/ehjdh/ztaa009 PMC9707905 36713963

[B46] van Opbergen C. J. M. Noorman M. Pfenniger A. Copier J. S. Vermij S. H. Li Z. (2019). Plakophilin-2 haploinsufficiency causes calcium handling deficits and modulates the cardiac response towards stress. *Int. J. Mol. Sci.* 20:4076. 10.3390/ijms20174076 31438494 PMC6747156

[B47] van Osta N. Kirkels F. Lyon A. Koopsen T. van Loon T. Cramer M.-J. J. (2021). Electromechanical substrate characterization in arrhythmogenic cardiomyopathy using imaging-based patient-specific computer simulations. *Europace* 23 153–160. 10.1093/europace/euaa407 33751081 PMC7943356

[B48] van Osta N. Lyon A. Kirkels F. Koopsen T. van Loon T. Cramer M. J. (2020). Parameter subset reduction for patient-specific modelling of arrhythmogenic cardiomyopathy-related mutation carriers in the CircAdapt model: parameter subset reduction. *Philos. Trans. R. Soc. A Math. Phys. Eng. Sci.* 378:20190347. 10.1098/rsta.2019.0347 32448061 PMC7287326

[B49] Voigt J. U. Pedrizzetti G. Lysyansky P. Marwick T. H. Houle H. Baumann R. (2015). Definitions for a common standard for 2D speckle tracking echocardiography: consensus document of the EACVI/ASE/Industry Task Force to standardize deformation imaging. *Eur. Heart J. Cardiovasc. Imaging* 16 1–11. 10.1093/ehjci/jeu184 25525063

[B50] Walmsley J. Arts T. Derval N. Bordachar P. Cochet H. Ploux S. (2015). Fast simulation of mechanical heterogeneity in the electrically asynchronous heart using the MultiPatch module. *PLoS Comput. Biol.* 11:e1004284. 10.1371/journal.pcbi.1004284 26204520 PMC4512705

[B51] Zenker S. (2010). Parallel particle filters for online identification of mechanistic mathematical models of physiology from monitoring data: performance and real-time scalability in simulation scenarios. *J. Clin. Monit. Comput.* 24 319–333. 10.1007/s10877-010-9252-2 20676923

