# Supplementary material for: Uncertainty Quantification of Regional Cardiac Tissue Properties in Arrhythmogenic Cardiomyopathy Using Adaptive Multiple Importance Sampling
Source: Front Physiol. 2021 Sep 30;12:738926. doi: 10.3389/fphys.2021.738926 (PMC8514656; doi:10.3389/fphys.2021.738926)
Supplement: Supplementary file 1 [file Data_Sheet_1.PDF]

Supplementary Material

1. Supplemental Methods

1.1. Adaptive multiple importance sampling: the proposal distribution

In order to increase stability and decrease the heterogeneity in sample weight, we first applied collapse prevention measures to the definition of proposal distribution. Second, we applied a transformation to draw samples along the principle components. Third, we applied the k-means clustering algorithm to generate smaller proposal distributions.

1.1.1. Proposal distribution and collapse prevention

Collapsed proposal distributions result in relatively high $q(\theta)$ values and low weights w. This means that the samples drawn from a collapsed proposal distribution will not contribute to the final distribution unless the total number of samples go to infinity. Collapsed proposal distributions should therefore be prevented. Each iteration, the proposal distribution $q_i(\theta)$ is based on the weights w of the drawn samples of all previous iterations[1]. To prevent the proposal distribution from collapsing, we propose a history based sample weight $w^{(\gamma)} \propto w^{\gamma}$ obtained with a power-law transformation and normalized such that $\sum w^{(\gamma)} = 1$. To ensure a sufficient effective sample size, γ was set such that the effective sample size is at least 10 times the number of parameters.

$$\gamma = \text{MIN}\ (\gamma|_{n_{eff}(w^{(\gamma)})=10\cdot n_{\theta}}, 1) \tag{S1}$$

In the extreme of $\gamma \to 0$, each sample has an equal sample weight of $w^{(\gamma)}_{sample} = 1/n_{samples}$ and for $\gamma = 1$, the sample weight is equal to the true sample weight $w^{(\gamma)} = w$. In the next steps, the gamma transformed sample weight is used to calculate the eigenvectors because the gamma transformation prevents it from being singular. The number of bins in the histogram $n_{bins,i}$ of parameter i used to determine the proposal distribution is defined using w^{γ} to prevent it from collapsing and is given by

$$n_{bins,i} = \left\lceil \frac{1}{2} \cdot \left(N_{eff}(w)\right)^{\frac{1}{3}} \cdot \frac{\text{MAX}(\theta_i) - \text{MIN}(\theta_i)}{IQR_i(w^{(\gamma)})} \right\rceil \tag{S2}$$

1.1.2. Sampling along the principal axis

A large sample size is essential to approximate the posterior distribution of interest. Due to computational limitations, the proposal distribution cannot be defined using a multivariate distribution. Alternatively, the samples were drawn from a univariate distribution for each principal component to include linear interactions and better estimate the distribution. Morzfeld et al.[2] used this for dimension reduction and sampled only along the principal axis of the most important eigen vectors. We do not use it for dimension reduction, but for spatial reduction of the proposal distribution.

The principal components are given by the Eigenvectors of the covariance matrix of the drawn samples θ. To include goodness of estimation while preventing the covariance matrix from being singular, the $w^{(\gamma)}$-weighted covariance matrix was used.

$$\theta = \mu + W \cdot x \tag{S3}$$

$$x = W^{-1} \cdot (\theta - \mu) \tag{S4}$$

with

$$W = eig\left(cov\big(\theta, weights = w^{(\gamma)}\big)\right). \tag{S5}$$

$$\mu = \sum w \cdot \theta \tag{S6}$$

Because W is an orthonormal rotation matrix, no correction is needed on the proposal distribution i.e. $q(\theta) = q(x)$.

The proposal distribution $q(\theta)$ is an approximation of the posterior distribution of the previous distribution and consists of multiple uniform distributions. This is obtained by making a histogram of the samples regarding its weight w. Each principal component is used independently of each other. To prevent the covariance matrix to become singular, $10 \cdot n_\theta + 1$ successful simulations were drawn from the initial proposal distribution $q_0(\theta)$.

1.1.3. Proposal distribution clustered with k-means

Sampling along the principal components only accounts for linear dependencies in the input space. To also include nonlinear behaviour, we defined multiple proposal distributions based on clustered samples. Based on the transformed samples x, samples are clustered using the K-means algorithm. Each cluster describes a proposal distribution with a univariate distribution for each principle component. The probability density function of the proposal distribution $q(x)$ is given by the sum of the clusters. To prevent the model from being dominated by one cluster in the first iterations, the minimum number of kernels was 5 and the maximum kernel weight was 0.25. The total number of kernels was maximum 100 and set by $n_k = \frac{n_{eff}}{2}$.

1.2. Likelihood in detail

The likelihood function is described as a function of the summed squared error X^2. This error is the sum of the weighted dimensionless errors based on strain ($e^2_{\epsilon,seg}$), strain rate ($e^2_{\dot{\epsilon},seg}$), inter-segmental strain differences ($e^2_{\Delta\epsilon_{inter}}$), ejection fraction (EF), LV end diastolic volume (EDV), and right ventricular diameter (RVD).

$$\mathrm{X}(\theta)^2 = \sum_{seg \in segments} \left(e^2_{\epsilon,seg} + e^2_{\dot{\epsilon},seg}\right) + \sum_{inter \in interseg} e^2_{\Delta\epsilon_{inter}} + \sum e^2_{other} \tag{S7}$$

The start time t_0 in measured strain was determined by the onset of the QRS complex. Because no ECG can be calculated from the CircAdapt model, there is no reference. An alternative to the QRS complex which is often used is mitral valve closure, however, this was not available in some datasets. To obtain a uniform definition of the likelihood function, the time t_0 in the model was defined as

$$t_0 = \arg\min_t \mathrm{X}(\theta)^2 \tag{S8}$$

The starting time t_0 in all time-series data such as strain were temporal shift to match the measured time-series data. Not all components in the likelihood distribution are t_0 dependent.

1.2.1. Strain and strain rate

Modelled strain in the myofibre direction was defined as the wall area relative to the wall area at onset:

$$\epsilon_{model,seg} = \sqrt{Am_{seg}/Am_{seg,t_0}} - 1 \tag{S9}$$

The error is calculated as

$$e^2_{segment} = \alpha_\epsilon \frac{1}{t_{end}} \frac{1}{n} \sum \left(\epsilon_{meas,seg} - \epsilon_{model,seg}\right)^2 \tag{S10}$$

Strain rate is defined as the time derivative of the strain. To reduce error magnification due to derivation, convolution is applied on both model and measurement strain rate.

$$\dot{\epsilon}_{convolve} = \dot{\epsilon} * f \tag{S11}$$

with f the convolution matrix. The error is calculated as

$$e^2_{strainrate,seg} = \alpha_{\dot{\epsilon}} \frac{1}{t_{end}} \frac{1}{n} \sum \left(\dot{\epsilon}_{meas,seg,convolve} - \dot{\epsilon}_{model,seg,convolve}\right)^2 \tag{S12}$$

The difference in strain is defined as

$$e^2_{\Delta\epsilon_{inter}} = \alpha_{\epsilon_{inter}} \frac{1}{t_{end}} \frac{1}{n} \sum \left(\left(\epsilon_{meas,seg1} - \epsilon_{meas,seg2}\right) - \left(\epsilon_{mod,seg1} - \epsilon_{mod,seg2}\right)\right)^2 \tag{S13}$$

The following segment differences are included: [RVapex, RVmid], [RVmid, RVbase], [RVapex, RVbase], and [LV, IVS].

1.2.2. EDV, EF, RVD

The last term in the likelihood function was defined as

$$\sum e^2_{other} = \frac{\left(V_{ed,meas} - V_{ed,mod}\right)^2}{\sigma^2_{V_{ed}}} + \frac{(EF_{meas} - EF_{mod})^2}{\sigma^2_{EF}} + \frac{(RVD - RVD)^2}{\sigma^2_{RVD}} \tag{S14}$$

1.2.3. Penalty functions

Adaptive importance sampling tends to explore areas in the input space that are not physiological, especially at higher temperature and in early iterations. Allowing this reduces its ability to converge. By adding penalty functions to the likelihood function, the model is enforced to produce physiological signals and more computational cost is focused on the area of interest. Indices included for penalty functions are, amongst others, a maximum mean left atrial pressure of 25 mmHg and a maximum right atrial pressure of 15mmHg to keep physiological pressures, and a maximum error on time to peak strain to enforce the systolic phase of the model to be in the window of interest.

1.3.Model parameters

The parameter subset θ consists of 20 parameters obtained from an extensive parameter sensitivity and identifiability analysis.[3] Included parameters including a description are shown in Table 2 of the manuscript. Samples are drawn using a log-uniform distribution or logit-uniform distribution to include boundaries. The log-uniform distribution includes only a lower-boundary LB. Samples are drawn from a proposal function $q^*(x)$ with $x_i \in [-\infty, \infty]$ and are translated to the probability density function in the normal domain following

$$\theta_i = \mathrm{LB} + e^{x_i} \tag{S15}$$

$$q_i(\theta_i) = \frac{1}{e^{x_i}} q_i^*(x_i) \tag{S16}$$

or generalized

$$q_i(\theta_i) = \left(\frac{d\theta}{dx}\right)^{-1} q_i^*(x_i) \tag{S17}$$

The logit-unform distribution includes both a lower boundary LB and a upper boundary UB. Transformation from $q^*(x)$ to $q(\theta)$ is given by

$$S(x) = \frac{1}{1 + e^{-x}} \qquad S^{-1}(x) = logit(x) = \mathrm{LOG}\,(\frac{x}{1 - x}) \tag{S18}$$

$$\theta_i = LB + (UB - LB) \cdot S(x_i) \tag{S19}$$

$$x_i = S^{-1}\left(\frac{\theta_i - LB}{UB - LB}\right) \tag{S20}$$

$$q_i(\theta_i) = S(x_i) \cdot \big(1 - S(x_i)\big) \cdot q_i^*(x_i) \qquad for\ \theta_i \in [-\mathrm{LB}, \mathrm{UB}] \tag{S21}$$

1.4.Pseudo code

Pseudo code 1: **importance sampling**

$i_{iter} = 0$	
$q_0 = U(lb, ub)$	
$\Theta = \{\}$	
while not converged:	
Draw $nSims$ samples θ and add to Θ	Pseudo code 2
Calculate $\mathrm{X}^2(\theta)$ for each sample	Pseudo code 3
Calculate probability density from proposal distribution $q(\theta)$	Equation (3)
Update proposal distribution $q_{i_{iter}+1}(\theta)$	Pseudo code 4
$i_{iter} = i_{iter} + 1$	

Pseudo code 2: **draw single sample**

For i_{par} in n_{par}	
Draw u from $U(0,1)$	
$k = K_i$ with $\sum_{j=0}^{j=i} w_i > u$ and $\sum_{j=0}^{j=i+1} w_j < u$	
Draw $x_{i_{par}}$ from k	
Transform x to θ	Equation (S3)

Pseudo code 3: **Calculate** X^2 **for each sample** θ

Run model $\mathcal{M}(\theta)$
If model is converged:
Get objective function $z = \mathcal{M}(\theta)$
Calculate $\mathrm{X}^2(z)$
Else:
$\mathrm{X}^2 = \infty$

Pseudo code 4: Update proposal distribution $q_{i_{iter}+1}(\theta)$

Determine T	
Calculate w and w^*	
Calculate $\theta_{opt} = \sum \theta \cdot w$	
Calculate principle components W	Equation (S5)
Transform each theta	
Define h_{max} and h_{min}	
$H = \{h_{min}, \ldots, h_{max}\}$ (10 steps)	
For i_{par} in n_{par}	
Histogram of w and w^* to θ_i with n_{bins} bin width b	
For = i_H in n_H	
$kmin = \min(Hist > H_{i_H}) - b$ and $kmax = \max(Hist > H_{i_H})$	
$kmin = \min\left(kmin, \theta_{opt,i_{par}} - 0.25 \cdot n_{bins} \cdot b\right)$	
$kmax = \max\left(kmax, \theta_{opt,i_{par}} + 0.25 \cdot n_{bins} \cdot b\right)$	
$K_{i_{par},i_H} = U(kmin, kmax)$	

1.5. Derived parameters

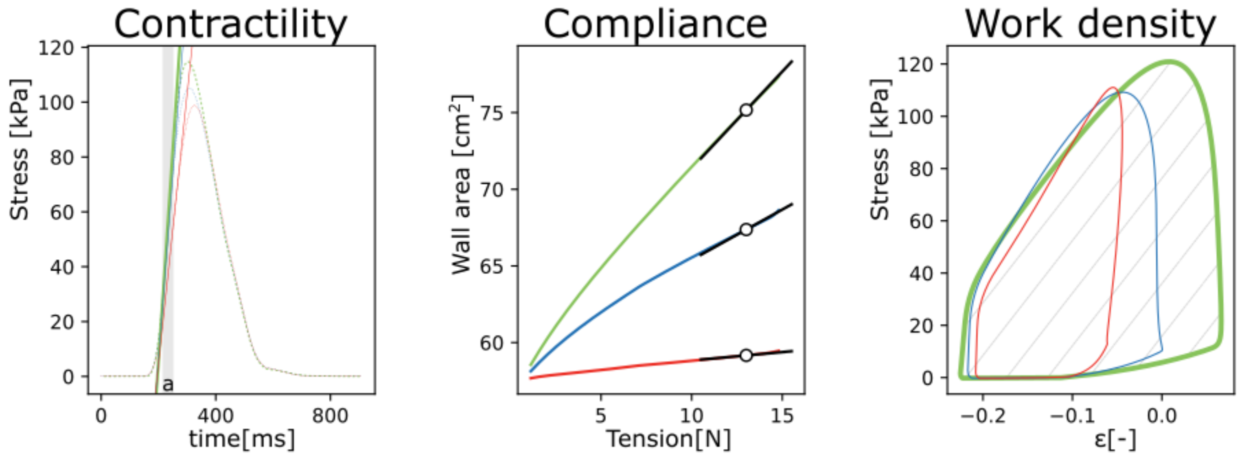

Figure S1: Derived parameters included in this study. (Left) Contractility is defined as the average stress-time derivative from 25% till 75% of the contraction curve (solid line in grey area *a*). (Middle) Compliance is defined as the wall area-tension derivative at end-diastole (black line around dot). (Right) Work density is defined as the area within the stress-strain loop.

2. Supplemental Results

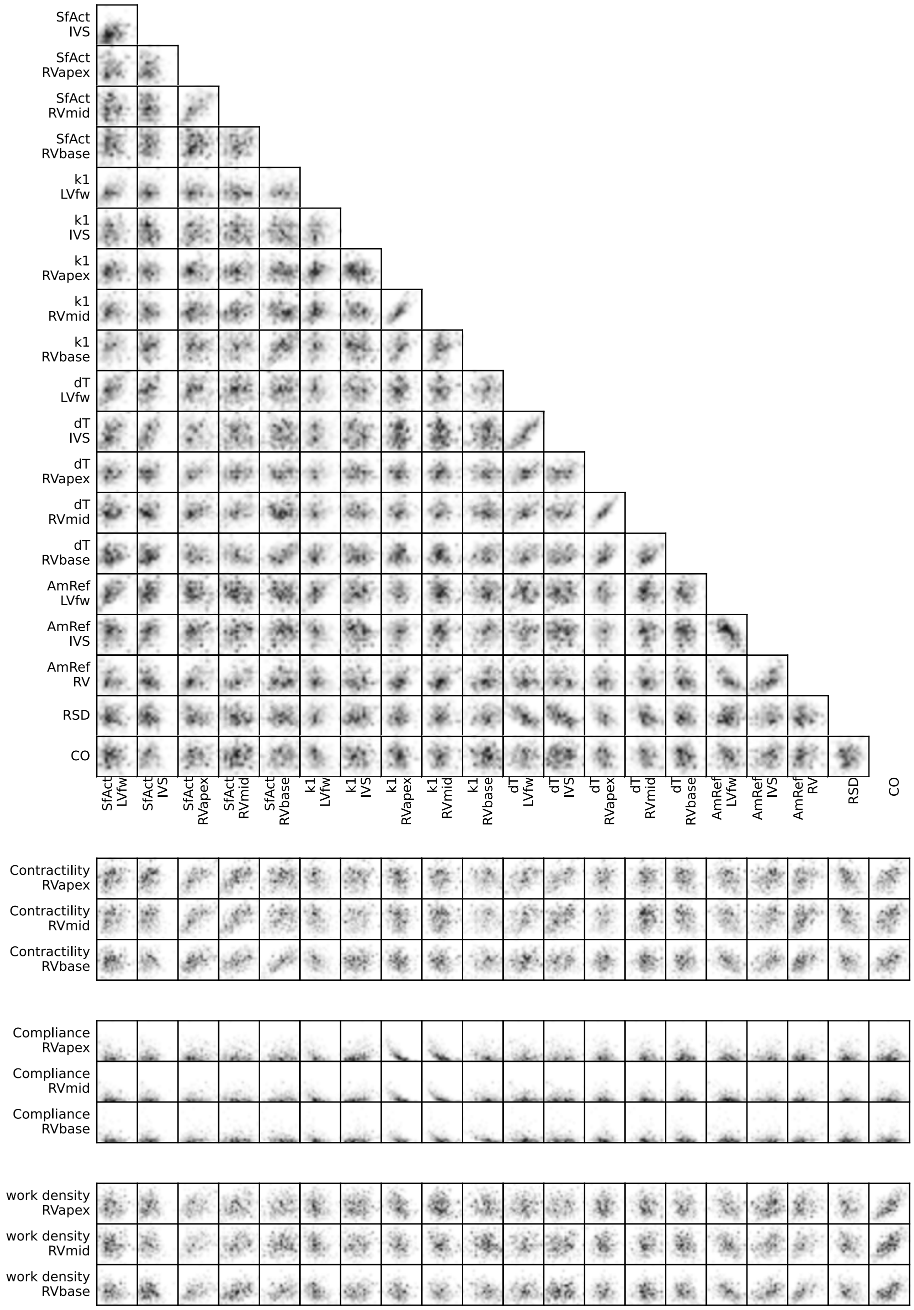

Figure S2: Posterior distributions of the estimated model parameters (top) and the correlation between the posterior distribution of model parameters and derived tissue properties (bottom). Axis are scaled to the minimum and maximum value of the distributions.

2.1. Reproducibility and Virtual Precision

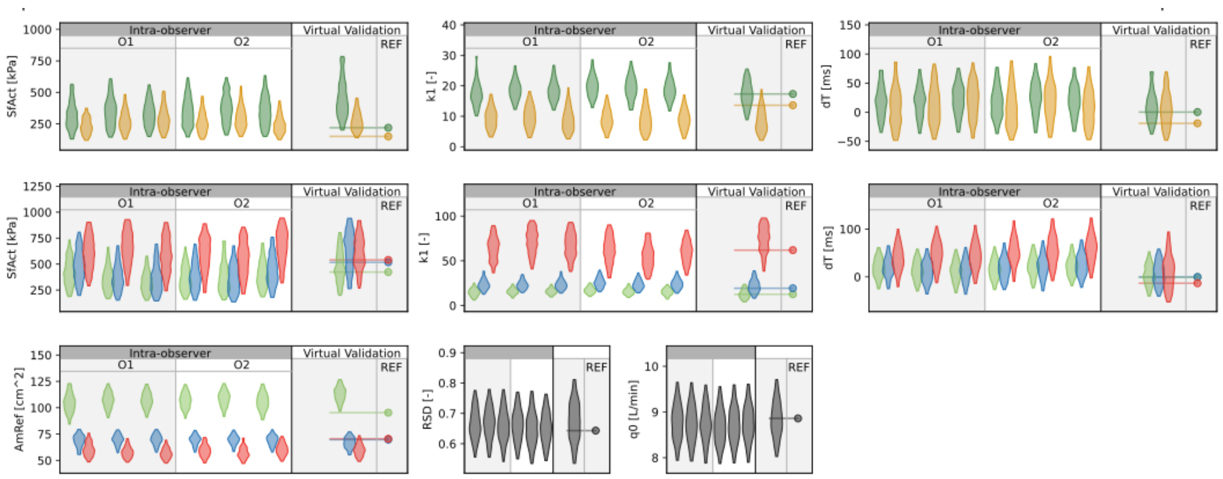

Figure S3: Intra observer validation and virtual validation. Two observations (O1 and O2) from the same observer are estimated 3 times each. From the most left distribution, a single simulation is used for virtual validation (dot on right). Colours match with the legend of Figure 1.

Table S1 Percentage of mutual information of the Cardiac Output distribution of patient 1. On the diagonal, the two squares show model reproducibility and on the cross-diagonal it shows the inter-observer variability.

	F1a	F1b	F1c	F2a	F2b	F2c	K1a	K1b	K1c	K2a	K2b	K2c
F1a		94.4	96.6	93.4	96.9	94.2	91.1	93.1	94.8	88.3	89.7	90.1
F1b	94.4		94.7	95.5	94.0	95.3	93.3	95.1	95.3	91.8	94.4	93.9
F1c	96.6	94.7		94.0	98.2	93.9	92.5	94.0	95.3	88.6	90.4	90.8
F2a	93.4	95.5	94.0		93.4	95.1	94.9	95.2	96.1	92.9	95.3	95.3
F2b	96.9	94.0	98.2	93.4		93.3	92.0	93.8	95.1	88.3	89.8	90.1
F2c	94.2	95.3	93.9	95.1	93.3		93.2	95.9	94.5	92.5	92.7	92.8
K1a	91.1	93.3	92.5	94.9	92.0	93.2		95.0	93.4	92.4	95.6	95.2
K1b	93.1	95.1	94.0	95.2	93.8	95.9	95.0		94.5	93.3	94.1	94.6
K1c	94.8	95.3	95.3	96.1	95.1	94.5	93.4	94.5		91.3	93.2	93.1
K2a	88.3	91.8	88.6	92.9	88.3	92.5	92.4	93.3	91.3		94.5	92.2
K2b	89.7	94.4	90.4	95.3	89.8	92.7	95.6	94.1	93.2	94.5		96.2
K2c	90.1	93.9	90.8	95.3	90.1	92.8	95.2	94.6	93.1	92.2	96.2	

Reproducibility
Interobserver
Intraobserver

Total Reproducibility: 94.5% (95%CI [92.5, 96.5])
Total Interobserver: 93.1% (95%CI [88.3, 95.9])
Total Intraobserver: 94.1% (95%CI [91.7, 97.6])

Table S2 Percentages of mutual information of all parameters in the first subject grouped by inter-, intraobserver and algorithm reproducibility.

	θ_0	θ_1	θ_2	θ_3	θ_4	θ_5	θ_6	θ_7	θ_8	θ_9	θ_{10}	θ_{11}	θ_{12}	θ_{13}	θ_{14}	θ_{15}	θ_{16}	θ_{17}	θ_{18}	θ_{19}	Total
Reproducibility	89.2	87.7	91.7	92.4	84.1	91.3	89.9	89.2	91.5	91.7	92.4	92.7	92.4	93	87.2	91.0	92.7	91.4	91.8	94.5	91.6 [74.6 – 96.2]
Interobserver	89.7	87.4	74.2	82.5	79.7	90.5	88.5	72.2	70	87.2	91.7	92.7	88.2	88.8	81.9	91.0	92.4	89.4	90.5	93.1	88.8 [51.8 – 95.3]
Intraobserver	87.8	87.5	68.5	78.5	65.3	90.1	84.8	69.0	55.4	85.4	91.2	92.6	86.9	83.7	73.1	91.0	93.2	83.7	88.9	94.1	87.2 [40.6 – 95.3]

Table S3 Percentages of mutual information of all patients grouped by Inter, Intra observer, and algorithm reproducibility. Median values with 95% CI are shown.

	Reproducibility	Inter-observer	Intra-observer
Subject 1	91.6 [74.7 – 96.3]	88.8 [51.8 – 95.3]	87.2 [40.6 – 95.3]
Subject 2	89.2 [66.4 – 96.2]	86.3 [64.8 – 94.8]	86.8 [62.3 – 95.3]
Subject 3	91.0 [65.9 – 95.8]	88.0 [42.3 – 95.6]	87.3 [54.0 – 95.5]
Subject 4	90.6 [72.3 – 95.8]	85.6 [46.3 – 95.2]	82.3 [27.8 – 94.3]
Subject 5	90.4 [58.3 – 95.6]	89.6 [56.9 – 95.8]	88.9 [59.2 – 95.7]
Subject 6	88.9 [65.9 – 95.4]	85.2 [53.0 – 94.5]	84.0 [50.0 – 94.3]
Subject 7	89.5 [64.6 – 95.5]	86.1 [50.2 – 95.0]	82.9 [50.8 – 94.8]
Subject 8	89.3 [65.3 – 95.3]	85.4 [55.9 – 94.3]	87.3 [63.4 – 95.3]
Subject 9	87.3 [24.6 – 96.1]	80.3 [16.0 – 94.5]	83.1 [19.9 – 95.0]
Total	89.9 [60.1 – 95.9]	86.5 [46.0 – 95.2]	85.9 [43.7 – 95.3]

Table S4 Highest density interval (HDI) percentage corresponding to the true value. The total HDI is 9% (95% CI [0 – 79]).

[%]	θ_0	θ_1	θ_2	θ_3	θ_4	θ_5	θ_6	θ_7	θ_8	θ_9	θ_{10}	θ_{11}	θ_{12}	θ_{13}	θ_{14}	θ_{15}	θ_{16}	θ_{17}	θ_{18}	θ_{19}
Subject 1	14	3	75	87	23	0	3	14	2	7	4	4	63	80	0	1	7	38	0	6
Subject 2	47	66	6	12	2	2	50	2	2	3	6	26	4	2	15	93	6	71	1	5
Subject 3	15	18	23	3	2	1	21	9	5	3	3	4	5	11	19	29	3	3	3	8
Subject 4	24	24	39	84	10	22	5	11	2	16	9	3	1	46	3	40	2	16	14	18
Subject 5	6	8	26	76	41	46	43	81	50	69	0	15	4	23	20	18	41	15	1	0
Subject 6	21	**99**	27	82	2	31	25	19	56	31	25	55	11	24	2	56	11	14	25	12
Subject 7	41	1	3	44	17	1	21	40	14	7	10	14	5	1	1	87	87	48	8	8

2.2. Additional plots

Figure S4-Figure S11 show additional results of two other patients. Patient 2 (Figure S4-Figure S7) had more abnormal strain with more pronounced pre-stretch and delayed onset-of-shortening compared to Patient 1. Patient 3 (Figure S8-Figure S11) had relatively more homogeneous strain compared to Patient 1.

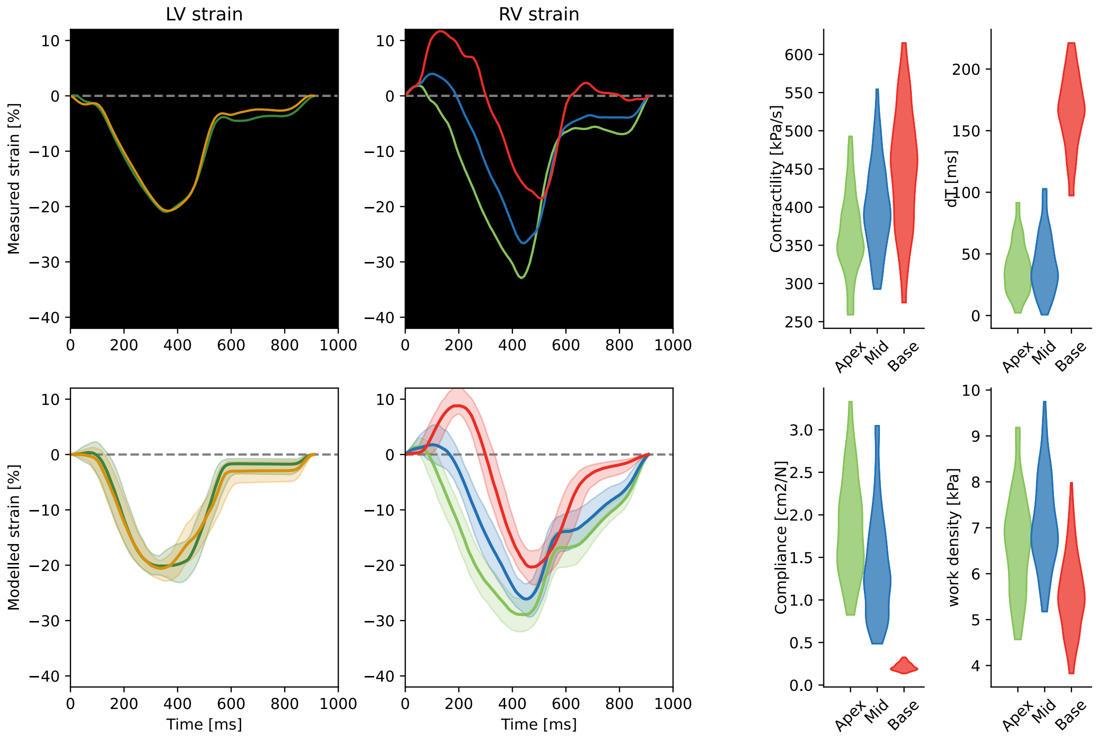

Figure S4: Example estimated strain (left) and derived parameters (right) of patient 2. This patient is used in the inter-intra observer study and virtual validation study.

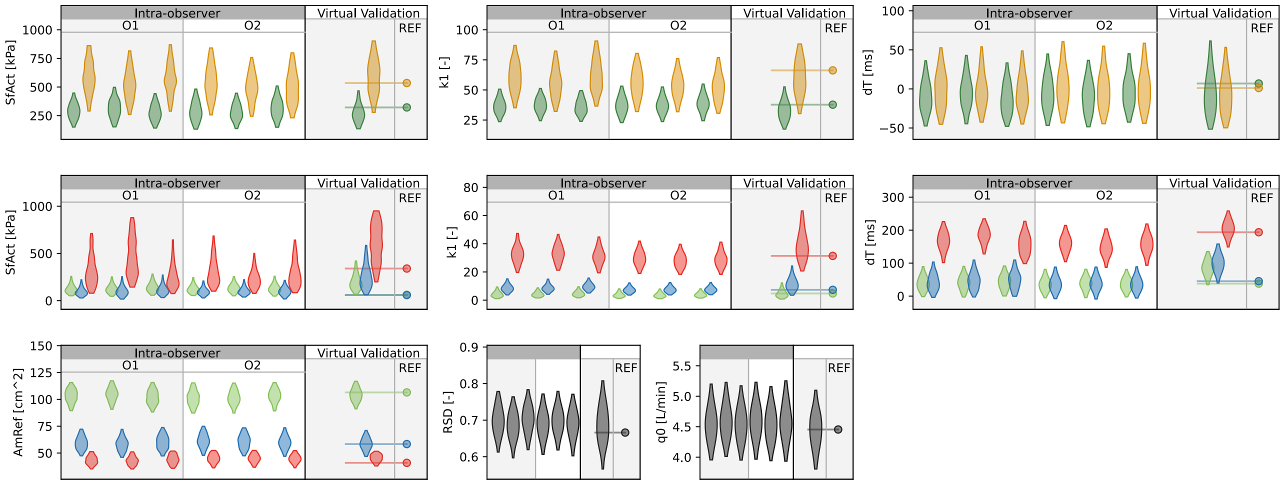

Figure S5: Intra observer validation and virtual validation of Patient 2. Two observations (O1 and O2) from the same observer are estimated 3 times each. From the most left distribution, a single simulation is used for virtual validation (dot on right). Colours match with the legend of Figure 1.

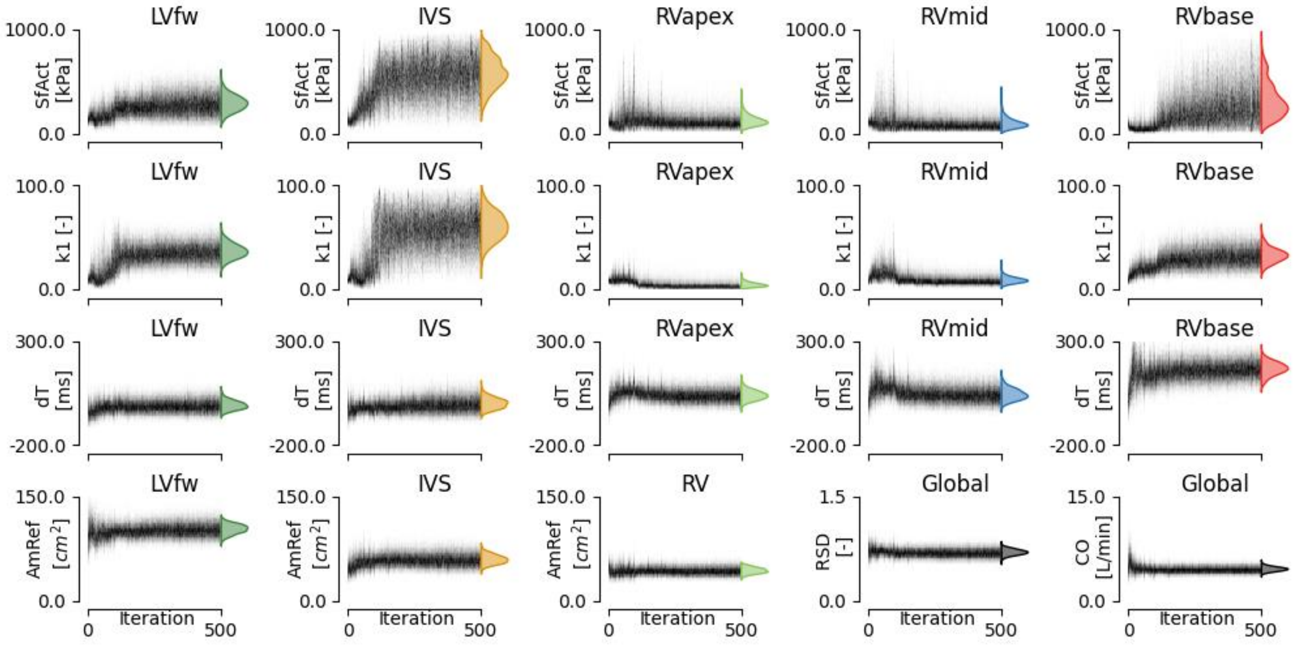

Figure S6: Convergence of estimated model parameters of Patient 2. The distributions on the right show the final estimated posterior distribution. Colours match with the legend of Figure 1.

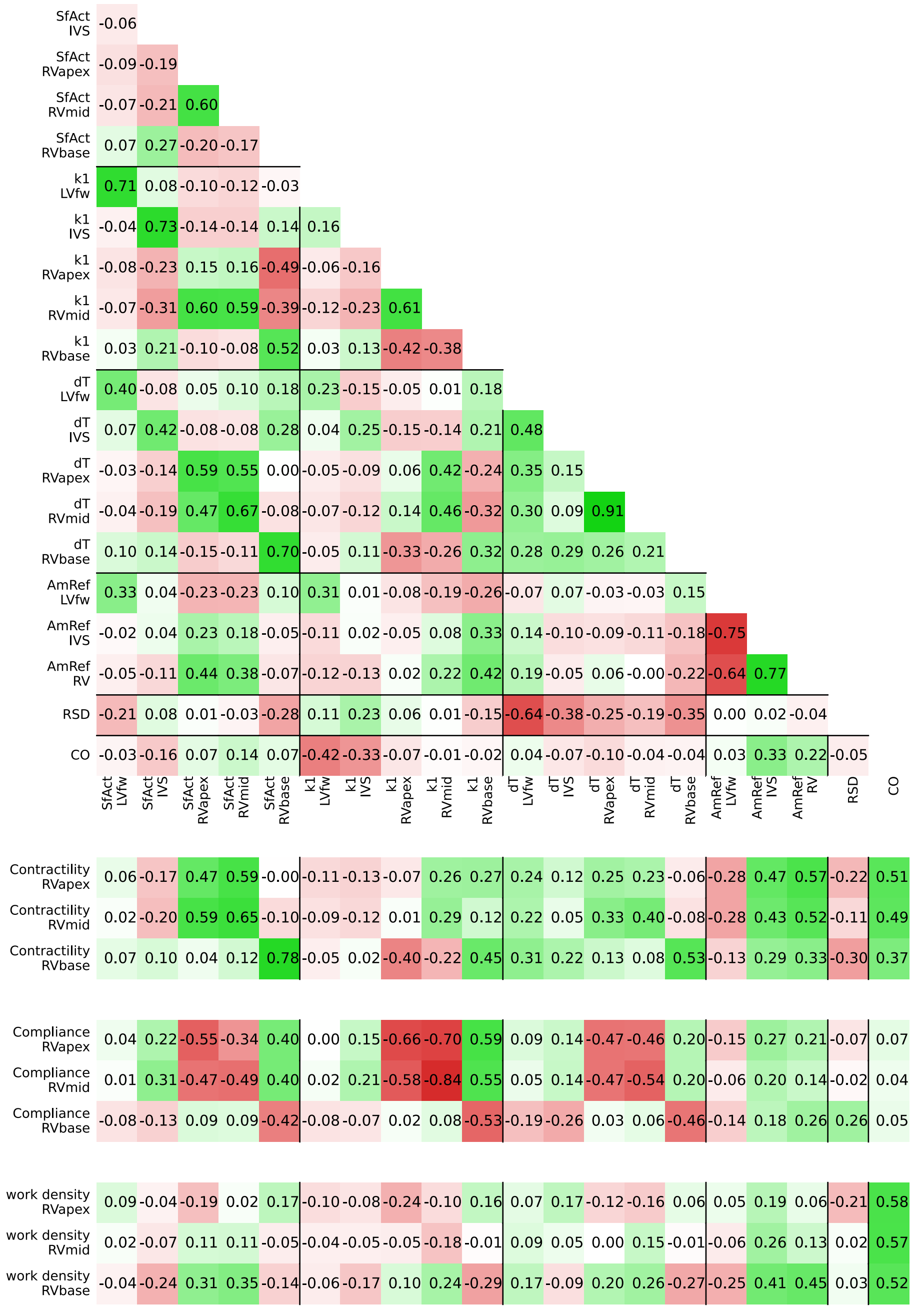

Figure S7: Posterior correlation matrix of the estimated model parameters (top) and the correlation between the posterior distribution of model parameters and derived tissue properties (bottom) of Patient 2.

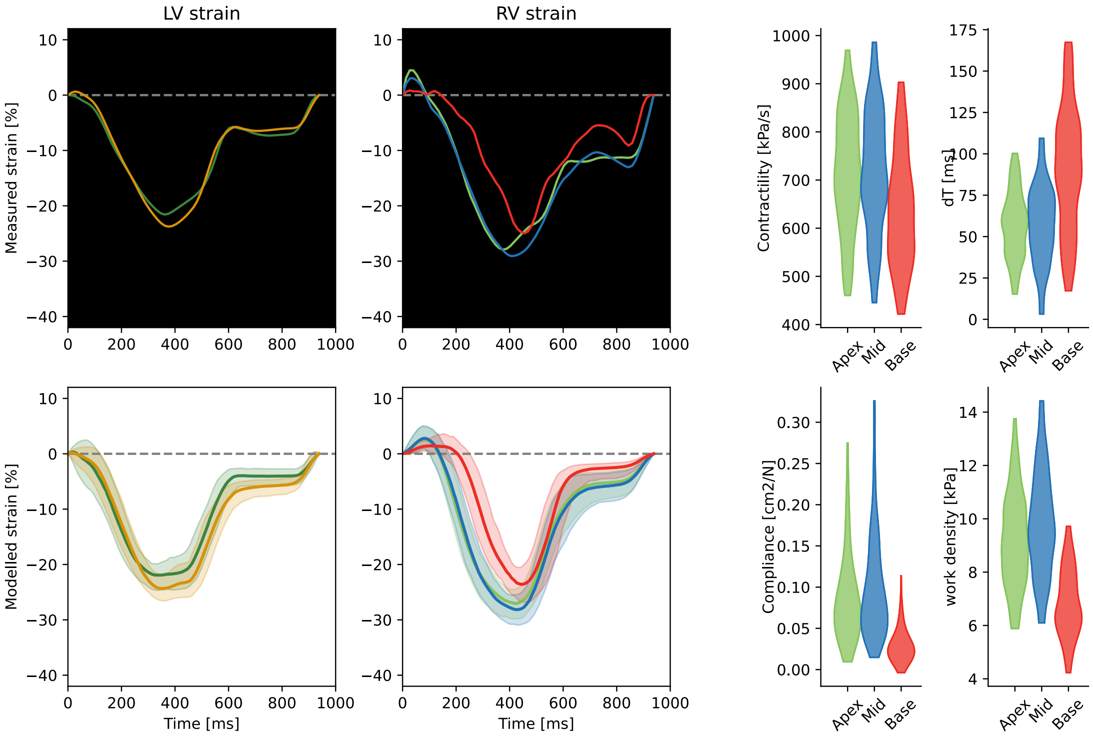

Figure S8: Example estimated strain (left) and derived parameters (right) of patient 3. This patient is used in the inter-intra observer study and virtual validation study. Colours match with the legend of Figure 1.

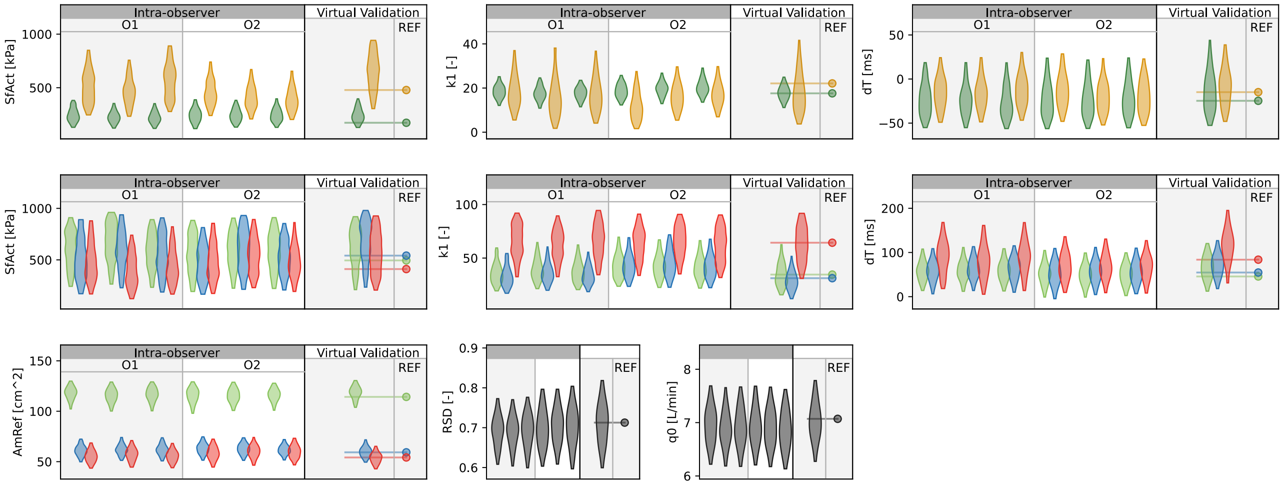

Figure S9: Intra observer validation and virtual validation of Patient 3. Two observations (O1 and O2) from the same observer are estimated 3 times each. From the most left distribution, a single simulation is used for virtual validation (dot on right). Colours match with the legend of Figure 1.

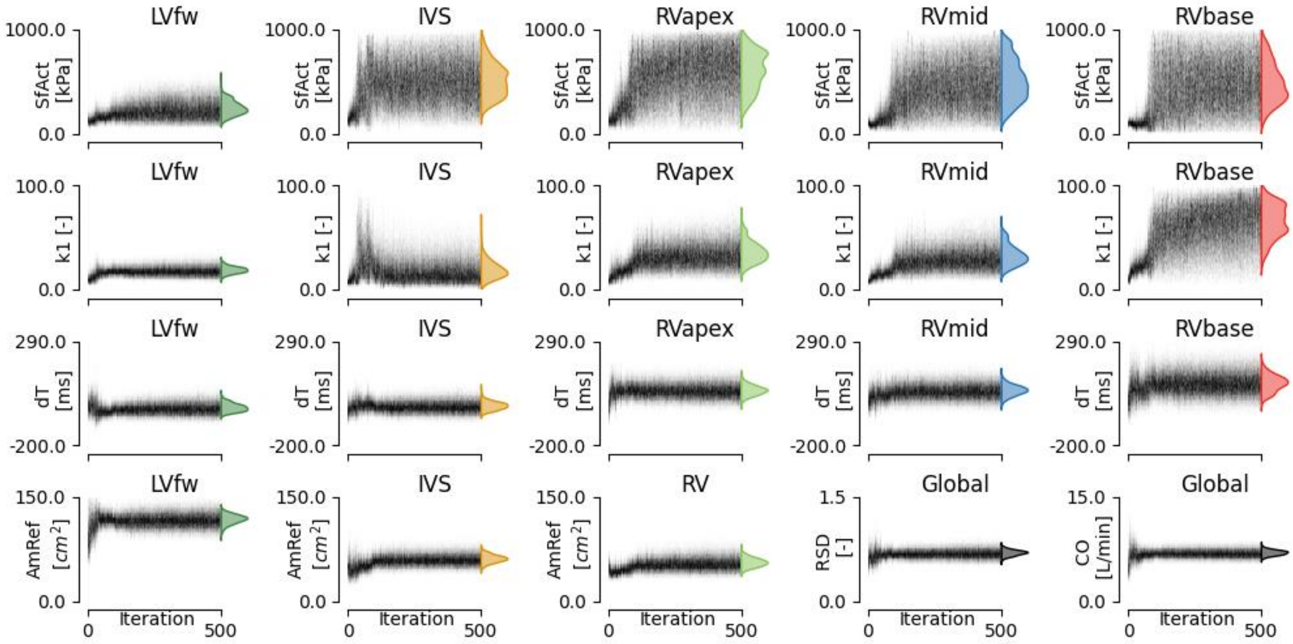

Figure S10: Convergence of estimated model parameters of Patient 3. The distributions on the right show the final estimated posterior distribution. Colours match with the legend of Figure 1.

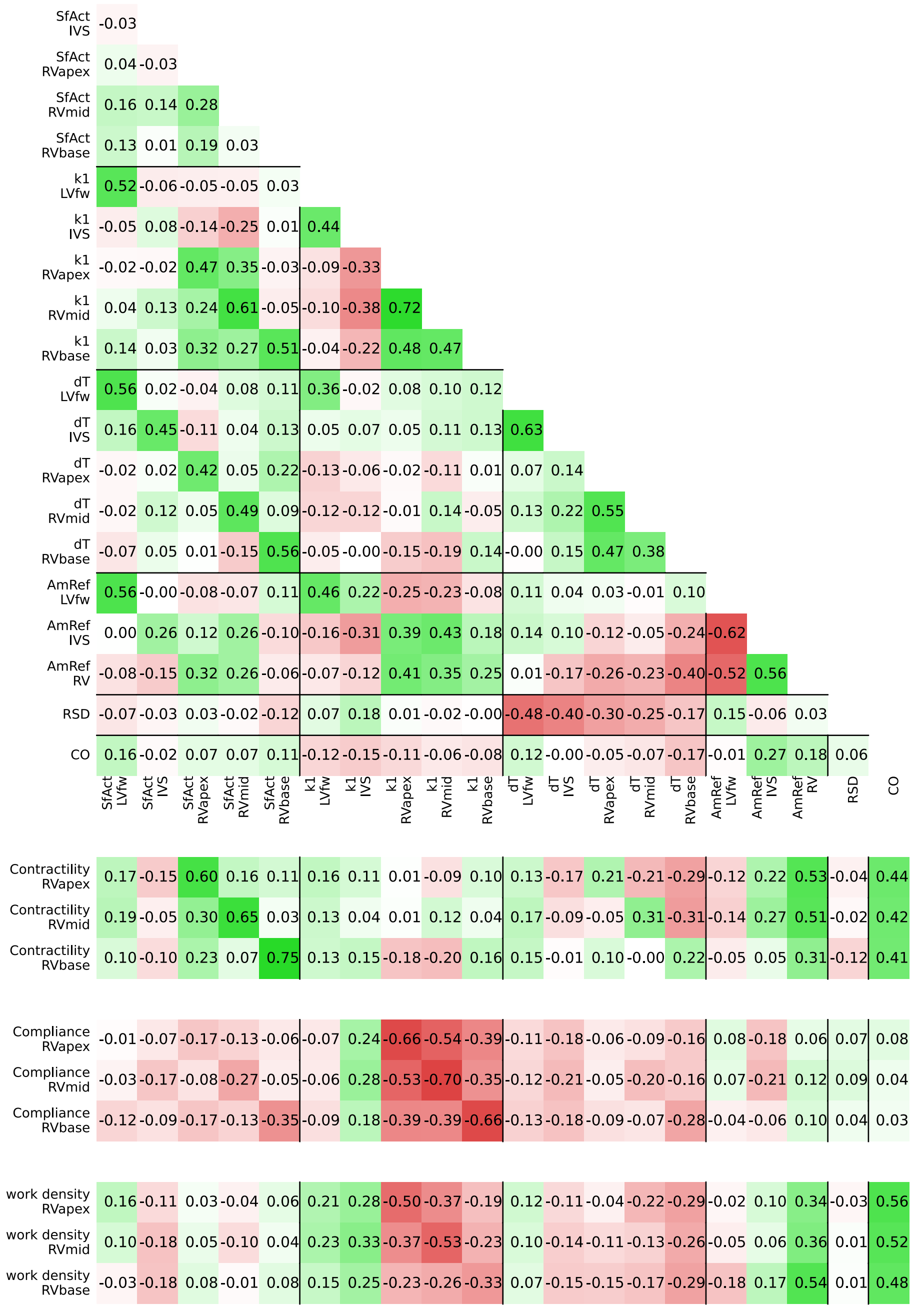

Figure S11: Posterior correlation matrix of the estimated model parameters (top) and the correlation between the posterior distribution of model parameters and derived tissue properties (bottom) of Patient 3.

References

1. Cornuet JM, Marin JM, Mira A, Robert CP. Adaptive Multiple Importance Sampling. *Scand J Stat* 2012;**39**:798–812.

2. Morzfeld M, Day MS, Grout RW, Pau GSH, Finsterle SA, Bell JB. Iterative importance sampling algorithms for parameter estimation. *SIAM J Sci Comput* 2018;**40**:B329–52.

3. Osta N van, Lyon A, Kirkels F, Koopsen T, Loon T van, Cramer MJ, *et al*. Parameter subset reduction for patient-specific modelling of arrhythmogenic cardiomyopathy-related mutation carriers in the CircAdapt model: Parameter Subset Reduction. *Philos Trans R Soc A Math Phys Eng Sci* 2020;**378**:20190347.
